# Exploring DNA movement through the application of droplet-based high-efficiency chromatin conformation capture (DropHiChew) and loop velocity

**DOI:** 10.1093/nar/gkag995

**Published:** 2026-10-29

**Authors:** Chen Zhang, Yeming Xie, Chen Tan, Zhichao Chen, Huimin Cao, Mei Guo, Luyang Zhao, Zhe Weng, Chong Tang

**Affiliations:** Shenzhen Loop Area Institute, Shenzhen 518000,China; Shenzhen Salus BioMed Inc. Ltd., Shenzhen 518000,China; BGI Genomics, Shenzhen 518083,China; BGI Genomics, Shenzhen 518083,China; BGI Genomics, Shenzhen 518083,China; BGI Genomics, Shenzhen 518083,China; BGI Genomics, Shenzhen 518083,China; Shenzhen Salus BioMed Inc. Ltd., Shenzhen 518000,China; NHC Key Laboratory of Male Reproduction and Genetics, Guangdong Provincial Reproductive Science Institute (Guangdong Provincial Fertility Hospital), Guangzhou 510600,China; Warshel Institute for Computational Biology, School of Medicine, The Chinese University of Hong Kong, Shenzhen, Longgang District, Shenzhen 518000,China; BGI Genomics, Shenzhen 518083,China

## Abstract

This study presents a novel approach to understanding DNA movement dynamics. Chromatin conformation plays a crucial role in controlling gene expression and cellular processes, with abnormalities linked to diseases like cancer and genetic disorders. ​Current single-cell chromatin conformation methods face limitations in cost-effectiveness, accessibility, and capturing dynamic genome architecture.​​ Therefore, we develop ​DropHiChew, a droplet-based, highly efficient chromatin conformation capture method employing 10× Genomics single-cell systems for user-friendly, cost-effective implementation. Combined with our ​loop velocity algorithm—which estimates the speed and direction of cell development by quantifying chromatin movement—this approach accurately gauges the trajectory of DNA motion and cellular states even at shallow sequencing depth (50k contacts per cell). Validation demonstrates its applicability across biological systems, including HEK293T, spermiogenesis, and embryonic development. The integration of DropHiChew and loop velocity offers potential for diverse applications in chromatin capture studies. This paradigm provides a dynamic perspective on genome organization for disease modeling, cellular differentiation, and developmental biology.

## Graphical Abstract

**Graphical Abstract:**
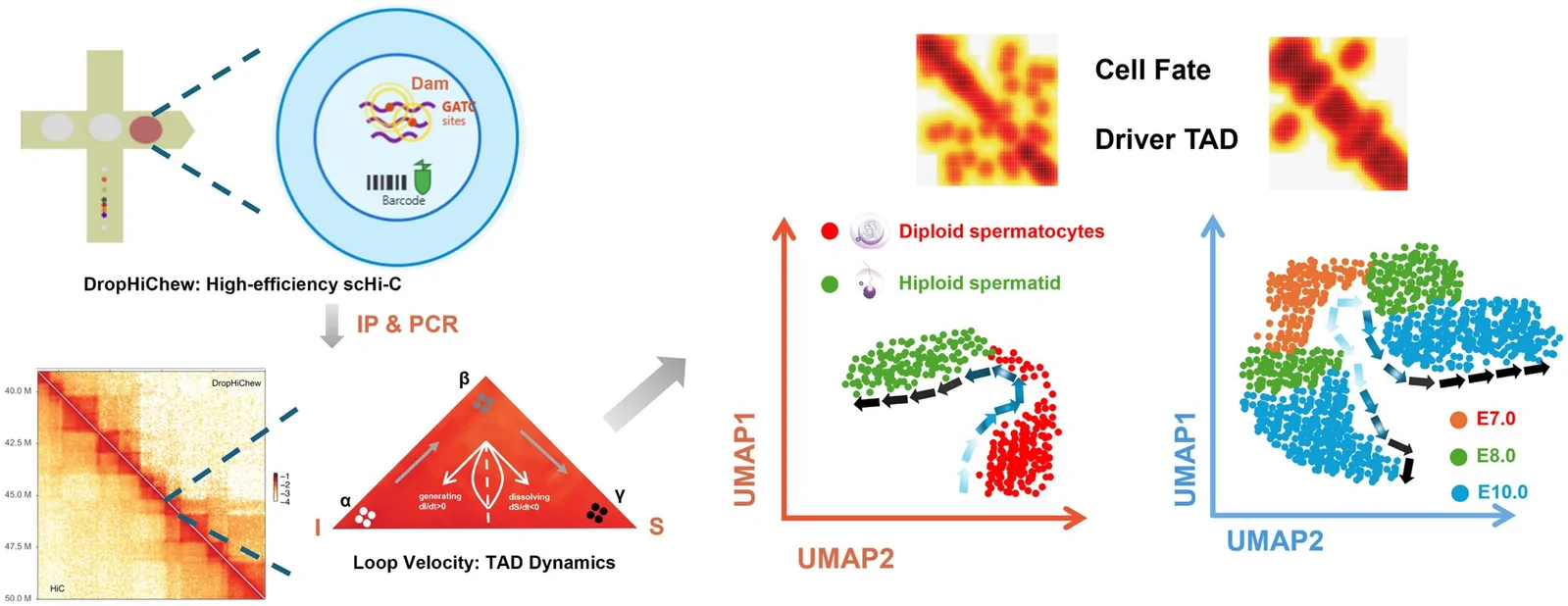

## Introduction

Chromatin conformation refers to the way DNA is arranged in three dimensions within the nucleus of a cell. It plays a crucial role in controlling gene expression and other cellular processes. Understanding chromatin conformation is important because it helps us understand how the genome is organized and how different regions of DNA interact with each other. Studying chromatin conformation is significant in the context of both disease and biological research. Abnormalities in chromatin conformation have been linked to various diseases, including cancer and genetic disorders [1]. By unraveling the complexities of chromatin conformation, researchers can gain a better understanding of the underlying mechanisms driving these diseases and potentially discover new treatment targets.

Scientists have been studying the dynamic folding of the genome within the cell nucleus over the years. It is been found that DNA is extruded into loop structures with the help of CTCF and cohesin [2]. This ongoing loop extrusion forms a more complex structure referred to as a topological association domain (TAD) [3]. The genome is then organized based on its proximity to the nucleus membrane, forming the constitutive lamin-associated domain and constitutive inter-lamin-associated domain. Notably, these domains have shown a strong correlation with the AB compartment in Hi-C experiments [4]. However, many of these structures are defined from a static perspective and average phenotype bulk sample. Given that DNA is highly dynamic within cells, we aim to observe the genome from dynamic viewpoints, which will greatly broaden our understanding of genomes. To investigate the dynamic actions of the genome, we require single-cell resolution to estimate movement in each cellular state.

Addressing a key challenge, scientists have created a method named single-nucleus Hi-C (scHi-C). This groundbreaking technique enables the examination of chromatin conformation at the level of individual nuclei, which helps reconstruct the 3D structure of chromatin. The first version of scHi-C featured biotin enrichment to amplify interactions, using a procedure similar to conventional Hi-C [5]. This involved genome digestion using a particular enzyme, adding biotin into the ends, and connecting proximal fragments. Paired-end sequencing was then applied to identify interactions between these fragments. It was later found, though, that biotin enrichment in single nuclei led to fragment loss, which impacted the resolution of observed chromatin conformations.

In response to this challenge, researchers developed a method known as Dip-C [6]. This approach eliminates the requirement for biotin incorporation and purification, enabling whole-genome sequencing without any DNA loss during the purification process. However, the solution does impact sequencing depth, resulting in over 90% of sequencing data being unutilized and only 8% of data providing valuable contact. In fact, most single-cell 3C techniques in common use today are based on the Dip-C concept. For example, Droplet Hi-C​ [7], which integrates an *in situ* Hi-C protocol with the 10× Genomics platform, enables ultra-high throughput​ and a rapid workflow​, while significantly improving data utility through barcoding within droplets.

Our laboratory recently developed snHiChew [8], a cutting-edge method using methyltransferase to label the ligation scar (GATC pattern) after polymerase chain reaction (PCR) in Dip-C, effectively boosting the efficiency to 45% valid pairs—currently the highest efficiency in single-cell chromatin capture technology. While impressive, these techniques require labs to design their cellular barcodes and apply the split-pool method for cell labeling. Therefore, we are on a quest for a more straightforward and cost-effective approach that can yield positive results for the majority of labs.

The advanced single-nucleus HiC (snHiC) technology developed in our lab has opened new avenues for investigating the dynamic structure of DNA across different cells and time points. Understanding cellular changes over time has been a long-standing interest among scientists. However, the majority of current detection technologies, such as single-cell RNA sequencing (scRNA-seq) and snHiC, provide only static snapshots of cellular states, lacking temporal context. Consequently, there is a crucial need to develop mathematical models that can predict dynamic cellular changes accurately.

RNA dynamic velocity has a long history and is well-researched [9]. Generally, our team [10] and others in the field use intron-rich RNA as an indicator of RNA synthesis and exon-depleted RNA as a sign of RNA degradation. This approach allows us to measure the rate of individual RNA production and degradation, which could be used to predict cellular destiny. Although using introns as indicators of nascent RNA speed is indirect and potentially biased [10], it is a necessary approximation at this point. However, the scientific community is continuously seeking to refine algorithms and methodologies for a more accurate estimation of nascent RNA expression [11–15].

In contrast to RNA velocity, measuring DNA movement directly reveals the spatial dynamics of distance. By using differential equations over time, we can estimate the speed of DNA movement with greater accuracy. Developing DNA velocity models allows us to interpret chromatin motion, offering a dynamic view of genome organization. This method not only improves our understanding of chromatin movement’s biological implications, such as transitions within AB compartments, but also provides dynamic parameters to describe the internal activity of TADs. Therefore, the DNA velocity algorithm is crucial for investigating the dynamic aspects of chromatin conformation in this field.

Our research is focused on finding practical and cost-effective ways to extend our knowledge of the DNA dynamic movement. We have used the 10× Genomics single-cell systems to develop a user-friendly, droplet-based chromatin conformation capture technique called DropHiChew. This efficient process involves digesting single nuclei with enzymes, ligating nearby fragments, attaching adaptor sequences using Tn5, and packaging single cells with barcode gel beads via the 10× Genomics Chromium system. After this, we carry out in-droplet reverse crosslinking and label each nucleus’s DNA with a barcode through linear amplification. Given that the 10× Genomics single-cell system usually yields thousands of cells, a budget of 2M per cell could be beyond the budget for many labs. To capture the movement of DNA in depth, we have crafted a new algorithm, loop velocity, based on loop extrusion models. Our research indicates that even with shallow sequencing (50k contacts per cells), we can accurately gauge the speed and direction of cell development using our loop velocity algorithm. We are confident that this method has great potential for diverse applications in future chromatin capture studies.

## Materials and methods

### Cell culture

We cultured both HEK293T, a human embryonic kidney cell line expressing a mutant SV40 large T antigen, and 3T3, a line of mouse embryonic fibroblasts, in Dulbecco’s modified Eagle’s medium (Gibco, no. 11965092). This was supplemented with 10% FBS (Fetal Bovine Serum, Gibco, no. 10099141) and 1 × penicillin-streptomycin (Gibco no. 15140122), and maintained at 37°C under 5% CO_2_. Spermiogenesis cells were prepared as our previous publication [16].

### Fixation protocol

The cells we collected were rinsed twice with cold phosphate buffered saline (PBS). We then cross-linked them in PBS containing 1% Formaldehyde (Thermo, No. 28908) for 10 min at room temperature. Afterwards, we neutralized the reaction with 250 mM glycine for 5 min at room temperature. Finally, we rinsed the cells twice again with cold PBS.

### Cell membrane penetration and chromatin decondensation

We resuspended the cell pellet in 500 μl of lysis buffer (containing 50 mM HEPES, pH 7.4, 1 mM ethylenediaminetetraacetic acid (EDTA), pH 8.0, 1 mM Ethylene glycol-bis(β-aminoethyl ether)-N,N,N′,N′-tetraacetic acid(EGTA), pH 8.0, 140 mM NaCl, 0.25% Triton X-100, 0.5% IGEPAL CA-630, 10% glycerol, and a 1 × protease inhibitor cocktail). We then let this sit on ice for 10 min before centrifuging it at 500 × *g* for 3 min at 4°C. The sample was then washed once with wash buffer (10 mM Tris–HCl, pH 8, 1.5 mM EDTA, 1.5 mM EGTA, 200 mM NaCl, 1 × protease inhibitor cocktail) and centrifuged again at 500 × *g* for 3 min at 4°C. We removed and discarded the supernatant, and gave the pellet one more wash with 1 × DpnII buffer.

The supernatant was carefully removed once more, and the pellet was then resuspended in 199 μl of 1.2 × DpnII buffer, inclusive of 0.1% sodium dodecyl sulfate (SDS). Following a thorough stirring, the pellet was treated in a 65°C metal bath for a duration of 10 min, swiftly followed by a 2-min cooling period on ice. The process concluded with the addition of 10 μl of 20% Triton X-100, accompanied by a 15-min incubation at 37°C with consistent agitation.

### Enzyme digestion and ligation

We started by adding 200 U DpnII (NEB, R0543L, final concentration 1 U/μl) to the previous reaction mix, giving it a good stir, and letting it react overnight at 37°C. Then, we added 2 μl of the digestion reaction mix to 7 μl NF water and 1 μl proteinase K, and let it all react at 65°C for an hour to break down the crosslinks. We checked the size of the DNA fragments using 1% gel electrophoresis.

Next, we deactivated DpnII by heating it at 65°C for 20 min. We then got rid of the supernatant through centrifugation and resuspended the pellet in 195 μl 1 × T4 DNA ligase buffer. We added 0.5 μl of 2000 U/μl T4 DNA ligase (ABclonal, RK21500) to achieve a final concentration of 10 U/μl. After a thorough mix, we let it react overnight at 16°C.

Finally, we took another 2 μl of this solution, added it to 7 μl NF water and 1 μl proteinase K, broke down the crosslinks at 65°C for 1 h, and confirmed the size of the bands using 1% gel electrophoresis.

Notably, the DropHiChew approach does not include an in-droplet ligation step; instead, ligation is carried out in bulk before droplet generation. To assess ligation efficiency, agarose gel electrophoresis was used for qualitative validation. Successful ligation was indicated by a shift in fragment size distribution compared to the digested sample, with the appearance of higher-molecular-weight products representing ligated DNA fragments.

### Chromium Next GEM single-cell ATAC

Nuclei were passed through a 40-µm (pluriSelect, no. 43-10040-40) and a 10-μm (pluriSelect, no. 43-10020-60) mesh filter. Afterwards, the instructions from the 10× Genomics Single Cell ATAC Operation Manual were adhered to.

The optimal cell-loading concentration was determined according to the Chromium Next GEM Single Cell ATAC Reagent Kits v1.1 User Guide. We first selected the target cell count per channel by referring to the Poisson look-up table provided with the kit. Subsequently, we prepared the cell suspension within the recommended input range and loaded the pre-calculated number of cells for each run. This standardized workflow ensured efficient droplet formation while minimizing the formation of doublets and empty GEMs.

### Methylation labeling and immunoprecipitation

The outcome of the previous procedure was amplified via PCR using new P5 and P7 primers to substitute the GTAC site and index at the P5 end with CATC or others (P5 5′- /phos/ AATGATACGGCGACCACCGACATCTACAC - 3′ P7 primers with phosphorothioate bond 5′ - CAAGC*AGAAGACGGCATACGAGAT - 3′). Next, we added 1 μg of DNA to the reaction, including 1 μl Lambda Exonuclease, 5 μl 10 × Lambda Exonuclease Reaction Buffer, and water up to 50 μl. This mixture was then incubated at 37°C for 30 min.

Afterwards, we purified the product and turned the remaining single strand into a double strand using a short RN1 primer (RN1 primer 5′ - TCGTCGGCAGCGTCAG - 3′, 95°C 3 min, 98°C 20 s, 58°C 1 min, 72°C 5 min by NEB Taq). We then added 500 ng of this purified, extended DNA to a reaction system composed of 5 μl 10 × *dam* Methyltransferase Reaction Buffer, 0.25 μl 32 mM SAM, 1 μl *dam* methyltransferase, and enough water to make up 50 μl in total. We incubated this at 37°C for 1 h to mark the GATC site m6A methylation.

Next, we treated the Protein A/G beads: we rinsed 10 μl Protein A/G beads twice with 1 × PBST buffer containing 0.1% Tween 20, then re-suspended them in 50 μl SuperBlock™ Blocking Buffer and incubated at room temperature for 15 min. We then washed the Protein A/G beads twice with 1 × IP buffer and re-suspended them in 48 μl 1 × IP buffer. We added 2 μl m6A antibody to this and allowed it to rotate at 4°C overnight.

Finally, we washed the Protein A/G beads pre-bound with antibody three times with 1 × IP buffer and re-suspended them in 50 μl 1 × IP buffer for later use. We diluted the purified *dam*-labeled DNA to 40 μl with EB buffer, denatured it at 95°C for 5 min, and immediately placed it on ice for 2 min. We then added 10 μl of pre-chilled 5 × IP buffer and 50 μl of pre-bound anti-m6A Protein A/G beads to the deformed DNA and mixed thoroughly. This was incubated at 4°C with rotation for 2 h.

We started by placing the beads on a magnetic stand and removing the supernatant. Next, we gave the beads a quick wash with pre-chilled medium stringency RIPA buffer. The beads then received two washes on ice with pre-chilled high-stringency RIPA buffer. After pre-chilling, we washed the beads once more with stringent RIPA buffer and gave them two final rinses with cold 1 × IP buffer.

We then resuspended the magnetic beads in 20 μl EB and added Qiagen protease to achieve a final concentration of 0.05 U/ml. The mixture was incubated at 50°C for 30 min and then at 70°C for 15 min.

Following this, we added 30 μl of a PCR mix (25 μl KAPA HiFi HotStart ReadyMix, 2 μl P5 primer, 2 μl P7 primer, 1 μl water) and ran 10 cycles of amplification on a PCR instrument. Finally, we purified the library with 1 × Ampure XP beads.

### Library circulation and sequencing

The library was prepared using the MGIEasy Circularization Kit (MGI, No. 1000005259), as per the kit instructions. The product was then processed with the MGISEQ-2000RS High-throughput Sequencing Kit (PE100) (MGI, No. 1000012554), for the creation of DNA nanoballs. These were sequenced using the PE100 + 100 + 10 + 16 mode on the MGISEQ-2000 platform and T7 MGI platform.

### Preprocessing of DropHiChew datasets

We began by sorting the raw reads for each cell with our in-house script. Then, we aligned single-cell Hi-C paired-end reads to either the HG19 or MM10 reference genome using HiC-Pro (v.3.1.0, default settings). This gave us HiC-Pro filtering and alignment metrics, valid pairs, and contact matrices. We corrected the matrices with ice_norm. We created contact matrices for each chromosome at resolutions from 10 kb to 1 Mb for more detailed analysis.

To estimate the number of valid cells in the DropHiChew data, we used a barcode rank plot to pinpoint the steep drop-off pattern that distinguishes valid cells from background noise. We plotted all detected DropHiChew barcodes in descending order by the number of nonduplicated valid pairs linked to each barcode. The R package kneedle helped us identify the transition between valid cells and non-cell barcodes. We then used the data associated with the valid cells for additional analysis.

### Comparison of pseudo-bulk chromatin architecture datasets

We prepared important metrics such as the *cis*/*trans* ratio, valid pair ratio, and valid pair dup, among others, from the HiC-Pro statistical outputs. The valid pair ratio is calculated by dividing the valid pairs (before duplication removal) by the reported pair from HiC-Pro. This method effectively illustrated the enrichment efficiency. On the other hand, the valid pair dup referred to the duplicated portion of valid pairs, as identified by HiC-Pro, providing an indication of the level of sequencing saturation.

To study the pattern of the contact matrix, we initially compiled the nonduplicated valid pairs from every cell and transformed them into mcool files with the help of the cooler tool (v0.8.2). Following this, we utilized the HiCExperiment (v1.2.0) R package to conduct a comparative analysis in pairs at various contact map resolutions. This analysis provides a deeper understanding of Hi-C features at the levels of the entire genome, specific chromosomes, TADs, and chromatin loops.

We evaluated the correlation of contact matrices at both chromosomal and TAD levels by utilizing genome-wide eigenvector scores (compartment score) and TAD insulation score, as determined by Cooltools (v0.4.1). Following this, the correlation was calculated using our own R scripts and the Pearson methods. To conclude, the contact distance decay curve was determined using HiCExplorer (v3.7.2).

### DropHiChew collision rate estimation

The data from the snHiChew HEK293T–NIH 3T3 mix were processed using a combined genome of HG19 and MM10. This was done using the standard snHiChew preprocessing methods. We utilized nonduplicated valid pairs in the cell rank plot to spot a sharp decline, identifying the empty cell barcodes. If a minimum of 89% of nonduplicated valid pairs were linked to HG19, we tagged the valid cell barcodes as HEK293T. We did the same for NIH 3T3, tagging them if 89% of pairs were linked to MM10. Any remaining valid cell barcodes were considered as doublets.

### Clustering of DropHiChew data in HEK293T–NIH 3T3 mixture data

We grouped the corresponding pairs related to the annotated HEK293T and NIH 3T3 cell barcodes. The dimensionality reduction calculations were carried out using Higashi [17], with standard settings in place. For Uniform Manifold Approximation and Projection (UMAP) clustering, we applied the first 15 principal components of the Higashi embeddings. The parameters were set to “n_neighbors = 5, min_dist = 0.01, metric=‘correlation’”.

### Comparison of single-cell chromatin architecture datasets

We compared how DropHiChew’s chromatin architecture data stacks up against data from snHiChew, Dip-C, and snHi-C. These are all well-respected single-cell chromatin conformation capture technologies. The datasets we used for this comparison were downloaded from the National Center for Biotechnology Information (NCBI) Gene Expression Omnibus (GEO), under the codes GSE94489 and GSE146397.

We utilized identical preprocessing and HiC-Pro metrics-based benchmark procedures for Dip-C, snHi-C, and snHiChew as we previously delineated. In order to evaluate sequencing yield efficiency, we reduced raw reads to diverse levels: 25k, 50k, 100k, 200k, 400k, 800k, 1.6M, 3.2M, 6.4M, and 12.8M. We strictly included cells whose read numbers surpassed these set thresholds.

Afterwards, we figured out the count of unique valid pairs per read for each threshold. We did this by fitting the data points to a saturation curve, using the model Y = B max * X/(Kd + X). To wrap things up, we gauged the read number for sequencing saturation for each single-cell chromatin conformation capture technology.

We examined the false positive rate by observing unexpected interactions between mitochondrial DNA and nuclear DNA. The false positive rates for each distinctive valid pair identified by HiC-Pro in every valid cell of the snHiChew and Dip-C datasets were calculated, as per the method outlined in Trac-looping [18].

### *In silico* HEK293T cell phasing over the cell cycle

We performed cell-cycle analysis using the method outlined in a previous study [19]. Essentially, we utilized the HEK293T 2-phase Repli-seq dataset (4DNESSV33VOL, 4DNESH4XLJCW) sourced from the 4D Nucleome Data Portal to label the early/late repli-score ratio for each cell. A higher early/late repli-score ratio signifies a cell closer to the early S-phase of the cell cycle.

### *In silico* testis cell phasing over the sex chromosome ratio

We performed an analysis of testis cell phasing, using the ratio of sex chromosome to autosome. Essentially, we labeled the sex chromosome ratio for each cell by dividing the number of fragments on chromosome X by the sum of chromosome X and chromosome 7 fragments. A sex chromosome ratio closer to zero signifies that the cell is more likely in the round or elongating spermatid phase of spermatogenesis.

### DropHiChew velocity input feature extraction

We took the pooled single-cell valid pairs, converted them into mcool files with a resolution of 25kb, and balanced them using cooler (v0.8.2). To identify pseudo bulk (pooled single cell) TAD boundaries, we utilized hicFindTADs from HiCExplorer (v3.7.2). We set parameters to “–minDepth 100 000 –maxDepth 750 000 –step 50 000 –thresholdComparisons 0.05 –delta 0.01 –correctForMultipleTesting fdr -p 20”.

Next, we quantified the 1D count of the inner 50k region for the left and right boundary of each pseudo-bulk TAD using bedtools intersect for each cell. We then normalized the 1D count for each region in accordance with the sequencing depth.

For TAD identification, valid cells were first downsampled to an equivalent number of non-duplicate valid pairs. The data were balanced and converted to .mcool format using Cooltools (v0.8.2) with the command –balance. TAD boundaries were subsequently called using hicFindTADs from HiCExplorer (v3.7.2) with parameters –minDepth 100 000 –maxDepth 750 000 –step 50 000 –thresholdComparisons 0.05 –delta 0.01 –correctForMultipleTesting fdr -p 20. This approach has been validated to produce robust boundary calls consistent with published methods [20].

### Theory of loop velocity

The model is shown in Fig. 2A. The theory of loop velocity is inferred from spindle-like dot plot of contacts with I boundary and S boundary. Loop velocity includes steady-state model and dynamical model using EM algorithm. The detailed inference and computational framework are elaborated in supplementary notes.

### Velocity analysis pipeline

RNA velocity was implemented in R package (velocyto.R) of original framework. All greedy balanced KNN algorithm in two samples used the default parameters. The velocity was estimated using gene.relative.velocity.estimates function with parameters “fit.quantile = 0.05, kCells = 10” from expression matrices of unspliced and spliced counts. The projection plot was drawn using show.velocity.on.embedding.cor function with parameters “show.grid.flow = TRUE, grid.n = 30, arrow.scale = 3.” Loop velocity was implemented in new R package—Loopvelocity (https://github.com/Dekayzc/Loopvelocity)—which contained steady-state model and dynamics model from matrices of contacts with I boundary and contacts with S boundary. All analysis related to loop velocity were included in Loopvelocity package.

The velocity field was calculated in the principal component space, a high-dimensional linear embedding derived from the chromatin contact data, and then projected onto the UMAP for visualization. This approach ensures that velocity calculations are performed in a distance-preserving linear space before non-linear dimensionality reduction for display purposes.

### Statistics

We carried out a two-sided *t*-test on the majority of the parametric data, adhering to a normal (or log-normal) distribution. For all hypothesis testing involving multiple TADs or genomic loci, Benjamini–Hochberg FDR correction was applied. In addition, we conducted a Pearson correlation analysis on this data. For non-parametric data or data that did not follow a normal distribution, we utilized the Wilcoxon rank test.

## Results

### Experimental design of DropHiChew

Droplet-based platforms like 10× Genomics and MGI DNBeLabC4 are leading the way in the single-cell field, bringing these experiments within reach for many labs. However, these platforms have not been used for single-cell chromatin conformation capture (3C) experiments yet, which makes single-cell 3C less accessible for most researchers. Considering the cost of sequencing the whole genome in Dip-C, we aim to enrich the valid pairs after PCR. This way, we can maintain a balance between capture sensitivity and cost-effectiveness (Supplementary Table S1). To achieve this, we plan on using *dam* methyltransferase to label ligation scars (GATC pattern, digested by DpnII in previous step), then enrich the ligation scar by methylation immunoprecipitation [8]. But there is a significant constraint when using this approach on commercial systems. The adaptor sequence or barcode sequence needs to avoid the GATC pattern, otherwise, these sequences will also be captured during the enrichment step, leading to a considerable barcode bias. According to single-cell barcode whitelist, 5% of 10× Genomics ATAC barcodes contain GATC, and 7% of MGI DNBeLabC4 barcodes contain GATC. This high prevalence of GATC-containing barcodes poses a challenge for implementing HiChew on commercial platforms.

We have developed a solution to this problem with our DropHiChew design (Fig. 1A). After *in situ* genome digestion by DpnII (GATC pattern) and proximity ligation (ligation scar–GATC pattern), the cells are transposed using the Tn5 adaptor in the kits (step 1–3). These cells are then loaded into the 10× Genomics platform with the scATAC-seq program (step 4).

**Figure 1.**
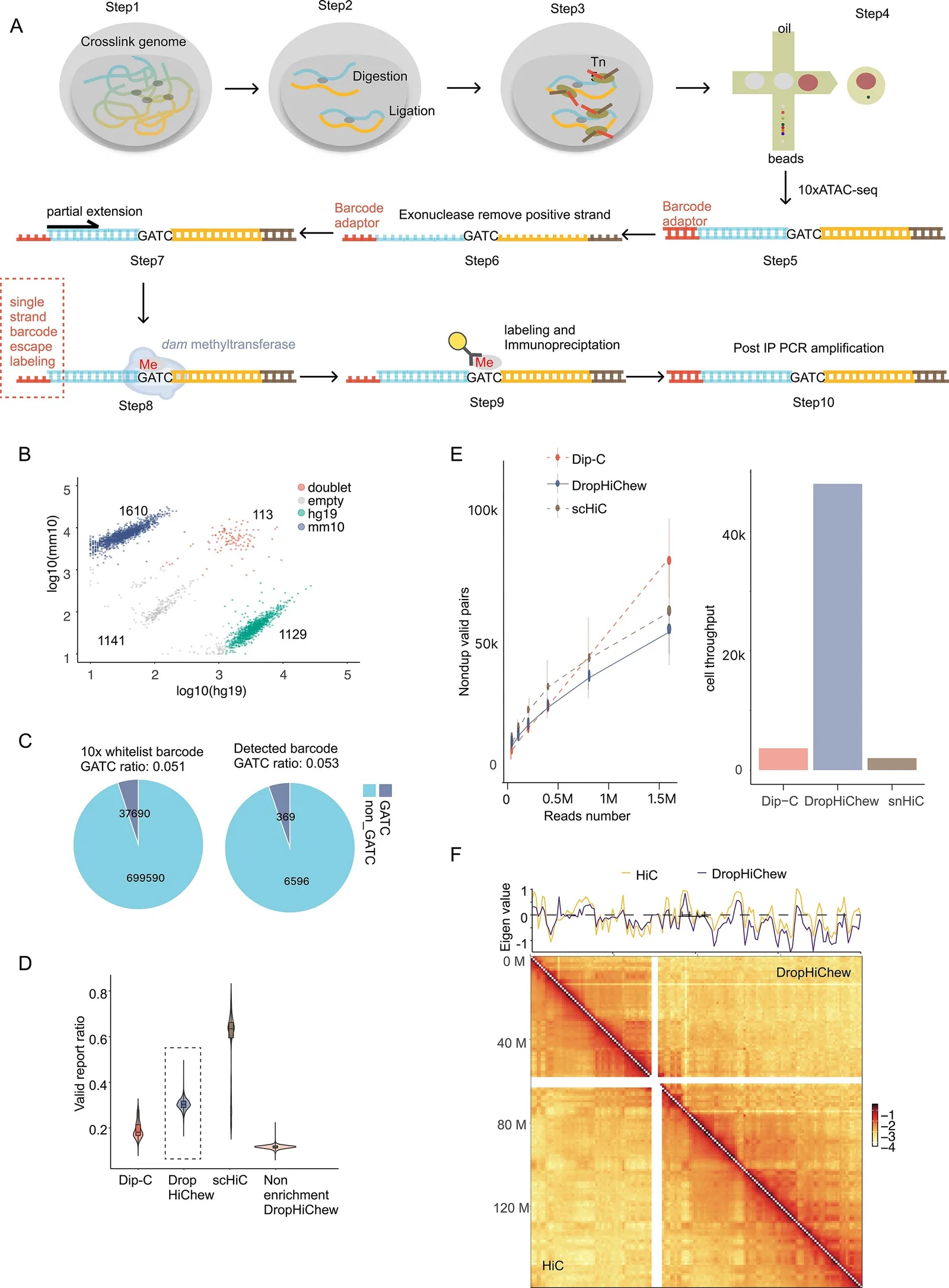
DropHiChew method development and evaluation. (**A**) The diagram provides an illustrated overview of the DropHiChew experimental process. (**B**) A combination of HEK293 and CHO cells was tested using the DropHiChew. The *X* and *Y* axes indicate the reads from the mouse and human genome in each cell, respectively. (**C**) A pie chart representation of the percentage of the GATC barcode included in the 10× whitelist, as well as the percentage of reads with GATC motif barcodes in the sequencing data, is presented. (**D**) The violin plot exhibits the valid report ratio (valid report pairs/reported reads) across different technologies. The term “non-enriched DropHiChew” pertains to the DropHiChew procedure absent of motif enrichment steps. (**E**) A line plot demonstrates the count of unique valid pairs (deduplication) across different methods at varying sequencing depths. The bar plot indicates the cell throughput under similar barcoding time. (**F**) The contact map (1M resolution) on Chr7 compares the DropHiChew and HiC. The upper line plot indicated the eigen value distribution on the genome.

Gel beads-in-emulsion (GEMs), which carry barcoded primers, encapsulate the cell within a droplet (Fig. 1A, step 4). A linear PCR amplification is then performed to attach the cell barcode primer to the adapted DNA fragments. Next, the emulsion is broken and barcoded DNA is pooled for PCR amplification to guarantee a sufficient amount of DNA (step 5).

We use a unique technique to increase the concentration of the 3C ligation scar (GATC) (Fig. 1A). This involves employing two universal primers in PCR, one with several phosphorothioate bonds and the other without. These bonds are resistant to exonuclease digestion. Subsequently, we use the Lambda exonuclease to break down the plus-strand DNA, leaving the minus-strand DNAs intact (step 6). Another primer that avoids the barcoding region is then used to extend on the single-strand DNA and create a double-strand DNA with a single-strand barcode at the end (partial extension) (step 7). The *dam* methyltransferase (GAmTC) can only act on the double-strand DNA, allowing the single-strand barcode to escape the methyltransferase modification (step 8). As a result, only the ligation scar with GATC can be modified with m6A methylation. We then carry out m6A methylation-specific immunoprecipitation to enrich the GATC ligation scar (steps 9–10). Finally, we assess the DropHiChew design to confirm if it successfully enriches the fragment with the ligation scar.

### Evaluation of the DropHiChew

In order to assess the effectiveness of DropHiChew on single-cell identification, we conducted a mix of HEK293T cells and mouse 3T3 cells in equal proportions (Fig. 1B). Upon final analysis, we identified 1129 cells as human and 1610 cells as mouse, with 113 cells classified as doublets. This doublet rate aligns with the 10X Genomics parameter of a 0.04 collision rate.

We have taken steps to refine our validation process by performing deeper sequencing of individual cells in HEK293T. This enhancement yielded an impressive 6864 cells per reaction, 100K valid contacts per cell, and 24K unique valid pairs (dedup) per cell (Supplementary Fig. S1A). We observed a duplication rate of 74% and a *cis*–*trans* ratio of 1.

Then, we turned our attention to the potential for a GATC bias in barcode detection due to GATC enrichment. Our analysis revealed that the GATC barcode accounted for 5%, consistent with the percentage of the 10× Genomics ATAC published whitelist (Fig. 1C). This suggests that the capture bias in DropHiChew is not significant.

We carried out a detailed analysis of the barcode containing the GATC motif to ensure that it does not skew the proportion of valid data. Our approach involved examining the valid pair ratio (valid pairs/qualified reads) across different barcode motifs. Our findings suggest that incorporating GATC into the labels could decrease the valid pair ratio using traditional methods (Supplementary Fig. S1B, left). This might happen as the GATC in the barcode and on the ligation scar GATC could compete for antibody-based enrichment. However, with DropHiChew, which omits barcode labeling, the difference was a mere 2%. This suggests that the efficiency of valid data stays relatively stable, regardless of the GATC’s presence in the barcode (Supplementary Fig. S1B, right).

The valid pair ratio is also a key metric for gauging an experiment’s success, showing how effectively the data are utilized. It is a handy tool for comparing the efficiency of various methods. We conducted a comparative analysis using several techniques, including snHiC, a biotin-based enrichment method, and Dip-C, a non-enrichment method. Notably, snHiC achieved an impressive average valid pair ratio of 63.4% (Fig. 1D). DropHiChew also delivered good results, with a ratio of 30.3%, while Dip-C registered an 18% valid pair ratio. However, DropHiChew without ligation scar enrichment, which is akin to Dip-C’s performance on the 10× Genomics platform, only reached an 11.7% ratio. It is important to highlight that the non-enrichment method could result in up to 88% of data being unutilized, underlining the importance of the enrichment steps.

Although our valid pair ratio is impressive, we must consider potential duplicate reads that may not be beneficial to our analysis. After eliminating these duplicates, we mapped the unique valid pairs across various sequencing depths of individual cells (Fig. 1E, left). The performance of DropHiChew is comparable to snHiC, producing valid pairs efficiently around 50K, marginally lower than the Dip-C at the 1.6M sequencing depth, which is sufficient for the subsequent study. While DropHiChew may not have outperformed the other two methods in unique contact yields, it is significant to note that DropHiChew offers the most direct and highest throughput for managing single-cell 3C experiments (Fig. 1E, right).

We have performed a thorough data quality assessment of DropHiChew’s performance by collating cell data into a simulated bulk sample and comparing it to the gold-standard HiC. We observed similar distance decay curves for both DropHiChew and HiC (Supplementary Fig. S1C). Upon evaluating various resolutions on the contact heat map, ranging from AB compartments to TAD, DropHiChew demonstrated a correlation of 0.82 and 0.77 in compartment eigen value and TAD insulation score distribution respectively (Fig. 1F and Supplementary Fig. S1D and E). In essence, the results from DropHiChew align commendably with the benchmarks established by conventional HiC experiments.

To contextualize DropHiChew’s performance, we performed a comprehensive comparison with published single-cell Hi-C methods using publicly available datasets. As shown in Supplementary Table S2, DropHiChew achieves a valid pair ratio of ∼30%, which is higher than Dip-C (∼18%) and comparable to snHiChew (∼45%) while offering significantly higher throughput than non-droplet methods. When comparing library complexity and cost-effectiveness, DropHiChew offers a superior balance for population-level single cell dynamics studies, with a cost per cell of ~$0.31 compared to $1.25 for Dip-C and $2.0 for sn-m3C-seq (Supplementary Table S1). These advantages make DropHiChew particularly suitable for large-scale studies requiring high cell numbers.

To further assess the robustness of DropHiChew across different experimental conditions, we evaluated the batch effect between two independent HEK293T preparations. The analysis revealed minimal batch effect between HEK293T-1 and HEK293T-2 replicates, with all key metrics showing between-batch to within-batch variance ratios below 0.11 (Supplementary Fig. S5). Furthermore, inter-batch correlation analysis demonstrated excellent reproducibility across independent experiments and operators, with valid pairs, valid interactions, and *cis* long-range contacts all showing high correlation coefficients (r > 0.85) (Supplementary Fig. S6).

In conclusion, DropHiChew presents a commercially viable and sustainable solution for managing large volumes of cells. We have structured this study with shallow sequencing in mind, given the substantial cell quantities. Additionally, the contacts change across cell of DropHiChew hints some regularity of DNA movement, which potentially allows us to observe dynamic genome structure changes. In subsequent research, we have devised a velocity algorithm which aids in understanding the dynamic DNA movements, even when using shallow sequencing.

### The loop velocity development and the TAD dynamic model

Recent studies [21–23] have shown that changes in TAD insulation or other structures during gene expression can offer valuable insights into chromatin motion during cellular development. However, even if the DNA does move, the speed cannot be simply determined by dividing the distance by time due to unknown time factors. Thus, the velocity of DNA movement could be estimated through dynamic models of DNA.

The Hi-C matrix allows us to visualize structures like TADs as beads in a 3D model, showcasing their high interaction frequencies. TADs are known for these frequencies, particularly their distinct boundaries created by significant interaction overlap in cells within these regions. These TAD boundaries often coincide with CTCF binding sites, sparking a lot of academic interest [24]. This correlation, backed by experimental findings [2, 25], led to the development of the loop extrusion model.

In the loop extrusion model, protein complexes like cohesin attach to DNA and initiate extrusion (Fig. 2A). The DNA passes through cohesin rings, forming a loop (Fig. 2A, Generating phase). The extrusion process halts on one side, at the DNA’s initiation boundary, which is blocked by CTCF binding. The initiation boundary (I boundary) stays in place, while the other boundary—the stabilizing boundary (S boundary)—moves toward the cohesin until a loop structure is created (Fig. 2A, Stabilizing phase). Once this process is complete, the cohesin unloads, leading to the disassembly of the TAD (Fig. 2A, Disassembling phase).

**Figure 2.**
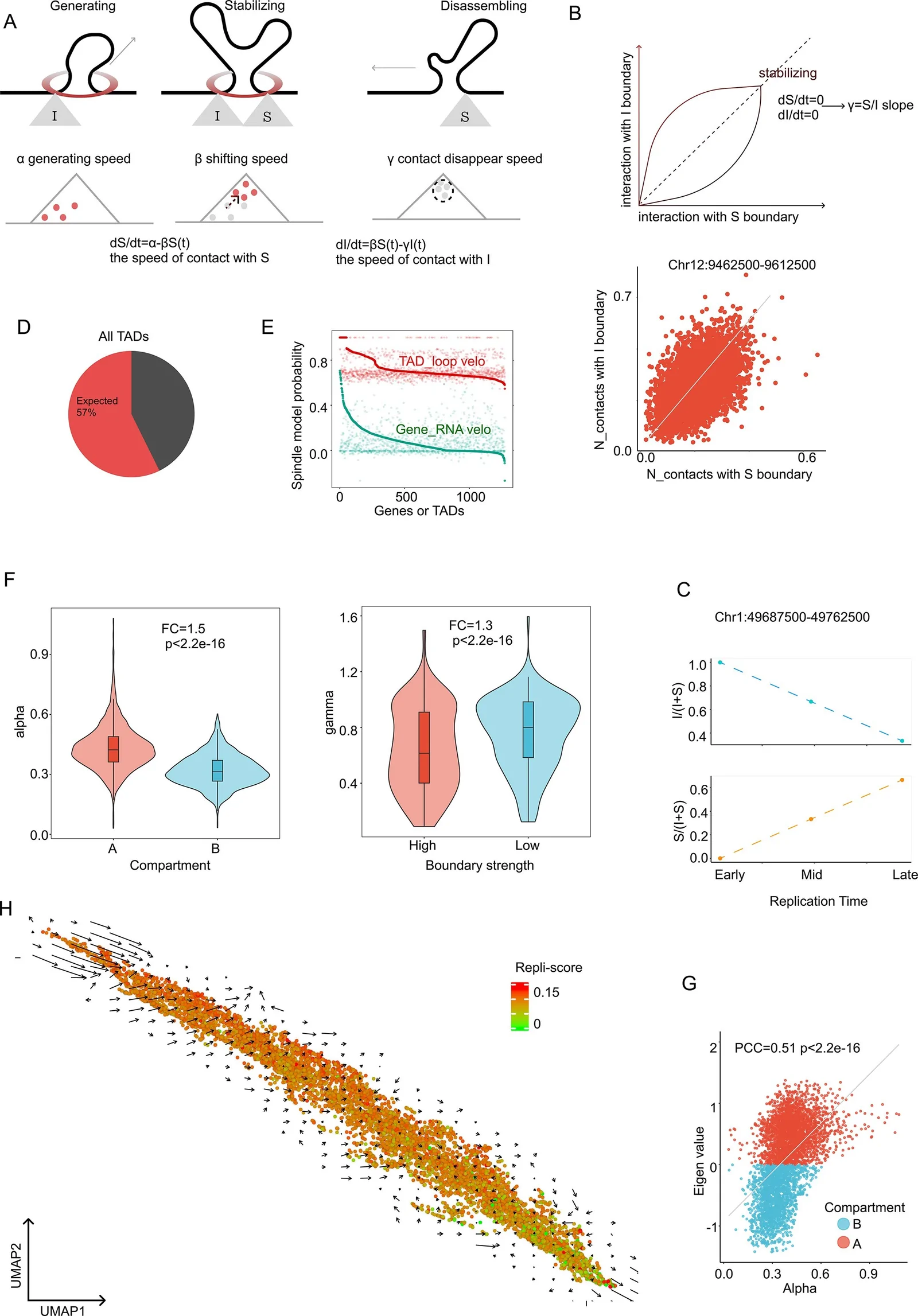
Loop velocity for HEK293 cells. (**A**) Illustration of our loop extrusion model alongside its mathematical counterpart. (**B**) A model we constructed to depict the interaction between the insulator (**I**) and stabilizer (**S**) boundaries, referencing a region from Chr3:4 203 750–42 062 500. The dot plot below represents individual cells, with axes indicating normalized interactions with S and I boundaries. The gray line depicts the overall trend of the I/S ratio across the cell population, conceptually corresponding to the dashed line in the theoretical model above. (**C**) A graph tracing the evolution of the average interaction ratio between the two boundaries across replication time, from early to late stages. The *y*-axis denotes the contact ratios of I boundaries (I/(I + S)) and S boundaries (S/(I + S)). (**D**) A pie chart revealing the proportion of TADs that conform to expected contacts change with loop velocity. (**E**) The rank curve illustrates the spindle model probability distribution of TADs in loop velocity (red line) and genes in RNA velocity (green line). The spindle model probability is determined by the Pearson correlation of the points, which indicates how close the points are to the regression lines. (**F**) The violin plots on the left and right represent the alpha value in the A/B compartment and the distribution of gamma, respectively, signifying both high (upper quantile) and low boundary strength (lower quantile). (**G**) A dot plot displaying the correlation between alpha and eigen value across all TADs. (**H**) A UMAP of the HEK293 cells, color-coded according to the repli-score, includes an arrow indicating the loop velocity.

As loop extrusion model described, a sequential and time-dependent process clearly indicate we can use a simple mathematics model, ordinary differential equations (ODEs), to quantify the time-dependent relationship between contacts with I boundary and contacts with S boundary. We assume that the rate of contact formation at the S boundary (or side) per unit time during loop extrusion is a constant, α (Fig. 2A). As the loop continues to wind, the contacts near the S boundary decrease at a rate we denote as β. At I boundary, the decrease in contacts at the S boundary corresponds to a potential increase of contacts at the I boundary, βS(t). Once the S boundary meets the cohesin anchored at the CTCF-blocked I boundary, a stabilized TAD is formed. After this, the cohesin is unloaded from the DNA, and the DNA continues to unwind into space, which we define as internal contacts decrease rate γ. Therefore, we can derive the following ODEs:


(1) \begin{eqnarray*}
\frac{{\mathrm{ d}S}}{{\mathrm{ d}t}} = \alpha - \beta S\left( t \right),\
\end{eqnarray*}

(2) \begin{eqnarray*}
\frac{{\mathrm{ d}I}}{{\mathrm{ d}t}} = \beta S\left( t \right) - \gamma I\left( t \right).\
\end{eqnarray*}

Here, I(t) indicates the number of contacts with the initiation boundary at time point *t*, and *S*(*t*) corresponds to the number of contacts with the stabilizing boundary at the same time point. The term d*I*/d*t* refers to the interaction with I boundary per unit time, while d*S*/d*t* denotes S boundary per unit time.

To solve unknown parameters α, β, and γ, the steady-state model can assist solution of some parameters. where d*I*/d*t* and d*S*/d*t* are zero, making these parameters solvable. The steady state was proposed when we analyzed a sample region (Chr12:9 462 500–9 612 500) to study the interaction distribution within each cell (Fig. 2B, bottom panel). The data suggest that these cellular processes contribute to forming a spindle model (Fig. 2B, upper panel). The intersection point represents the stabilization phase, similar to the steady state seen in the RNA model. During this steady state, the DNA loop extrusion and winding process halts, and the extrusion speed relative to the two edges approaches zero (d*I*/d*t* = 0 and d*S*/d*t* = 0) (Fig. 2B). We set β to 1, meaning we measure all rates in terms of the S boundary contacts decrease rate. Then, the contacts with I boundary and contacts with S boundary are constrained to a linear relationship where *S* = γI. So, γ can be solved through least square linear fit. After all the parameters are solved, we can estimate the subsequent cellular state through velocity calculation.

To summarize, the derivation of the complete equation and parameters can be found in the supplementary notes. This velocity model, named Loop velocity, uniquely utilizes Hi-C TAD features and incorporates the loop extrusion concept.

### Perform the loop velocity on the HEK293T cells from DropHiChew

In our study, we applied the loop velocity algorithm to HEK293T cells to validate our model. Our investigation identified 3333 TADs within these cells. Upon examining the I_S dot plot for loop velocity models across different regions (Supplementary Fig. S2A), we found that 57% of TAD regions could conform to expected trend of contacts over time with loop velocity (Fig. 2D). Most of the TADs exhibited a spindle model probability of >0.8, suggesting a strong resemblance to our mathematical models (Fig. 2E). This is a substantial improvement over RNA velocity, which typically shows spindle probabilities <0.4. The limitations of RNA velocity stem from its dependence on short-read sequencing from 10× Genomics scRNA, limiting its ability to capture intron reads in RNAs. As mentioned before, this could significantly underestimate introns and prevent certain genes from forming the spindle-like model, possibly skewing RNA velocity predictions [10].

We also examined whether DNA motion depends on time, forming the basis of our theory. We use replication scores to signify time within HEK cells, showing the shift from early to late replication stages. Next, we calculate the average interaction distribution at different time frames within the initiation and stabilization boundaries of selected TADs (Fig. 2C and Supplementary Fig. S2B). We observed an opposite trend at both boundaries correlating with replication states, underscoring a significant connection between interaction movement and developmental time.

We then assessed the two parameters, α and γ, in loop velocity model for each relevant TAD. Our model suggests that a higher α value points to a quicker establishment of contacts, while a heightened γ value signifies a faster pace of TAD dissolution. Additionally, we delved into the possibility of α and γ being associated with genome organization aspects.

To provide an intuitive biological interpretation of these parameters, we note that the parameter α (with units of contacts per second) represents the rate at which new contacts are formed at a boundary due to loop extrusion. Based on single-molecule studies, condensin extrudes DNA loops at speeds of up to ∼1.5 kbp/s. The product *α* × *L* (where *L* = 50 kb is the genomic window size) provides an estimate of the average physical DNA extrusion speed in base pairs per second. For instance, if *α* = 0.016 contacts/s, the corresponding extrusion speed would be 0.016 × 50 000 = 800 bp/s (0.8 kbp/s), consistent with experimental measurements (0.1–1.5 kbp/s). Thus, α serves a dual purpose: it quantifies contact change rates in Hi-C data and can be translated into biophysically meaningful loop extrusion speeds.

Our research suggests that TADs in the A compartment typically exhibit a significantly higher α value compared to those in the B compartment (Fig. 2F). A global correlation of 0.4 between alpha values and eigenvalues underscores this relationship (Fig. 2G). Furthermore, TADs with high chromatin accessibility and other markers of active histone also present significantly higher α values, aligning with the established correlation between eigenvalue and active transcription (Supplementary Fig. S2C). This leads us to infer that α, indicative of TAD generation speed, may have a close association with eigenvalue and chromatin activity.

Interestingly, we observed that γ, representing the speed of TAD dissolution, delivered contrasting results. It was considerably lower in the A compartment and active histone markers, albeit not as significantly as alpha’s correlation (Supplementary Fig. S2C). The most robust correlation was seen with boundary strength, a factor that depicts TAD stability (Fig. 2F). A correlation of −0.2 suggested that TADs with higher boundary strength dissolve more slowly, which meets our expectation. Further exploration of the boundary strength–related factor, CTCF, revealed a negative association with the γ factor (Supplementary Fig. S2C). This implies that the rate of TAD dissolution (γ) has a weak negative relationship with both CTCF signal and boundary strength.

Once we have figured out the α and γ for each TAD, we will check if the velocity we have calculated can forecast the genome organization’s next virtual time point. We then condensed this information into a 2D UMAP to highlight the upcoming time point for the cell state. Within this UMAP plot, we will use color gradients to denote replication scores, transitioning from early to late replication stages (Fig. 2H). We then estimated the expected velocity direction and compared that with the actual direction in real cells [11]. Our findings showed a significant correlation, with >60% of the predicted movement directions lining up with what we expected (Supplementary Fig. S4A). This suggests that the α and γ can effectively capture and predict the state of biological processes. To ensure that the loop velocity parameters are not sensitive to sequencing depth, we performed a comprehensive sequencing depth analysis. Both alpha and gamma parameters remained highly stable across different sequencing depths, with correlations exceeding 0.9 when the depth reached 100 million reads or more (Supplementary Fig. S7).

In conclusion, our studies indicate that our dynamic models, integrating loop velocity, can efficiently predict cellular development direction in cultured cells. These parameters with velocity could provide a more dynamic representation for TAD.

### Perform DropHiChew for spermiogenesis cell in testis

RNA velocity has gained acceptance due to its translation activity. The key question for loop velocity is understanding its role in driving phenotypes. To examine how loop velocity impacts cell differentiation over time, we selected the mouse testis spermiogenesis model due to its rapid phenotype changes. This model also conveniently omits the mitosis process, which allows us to study loop velocity’s influence on spermiogenesis without the complication of cell cycle-related genome compaction. An added benefit of utilizing spermiogenesis cells is their unidirectional cell differentiation. They transition from diploid cells, like pachytene spermatocytes, into haploid cells such as round spermatids and elongating spermatids (Fig. 3A).

**Figure 3.**
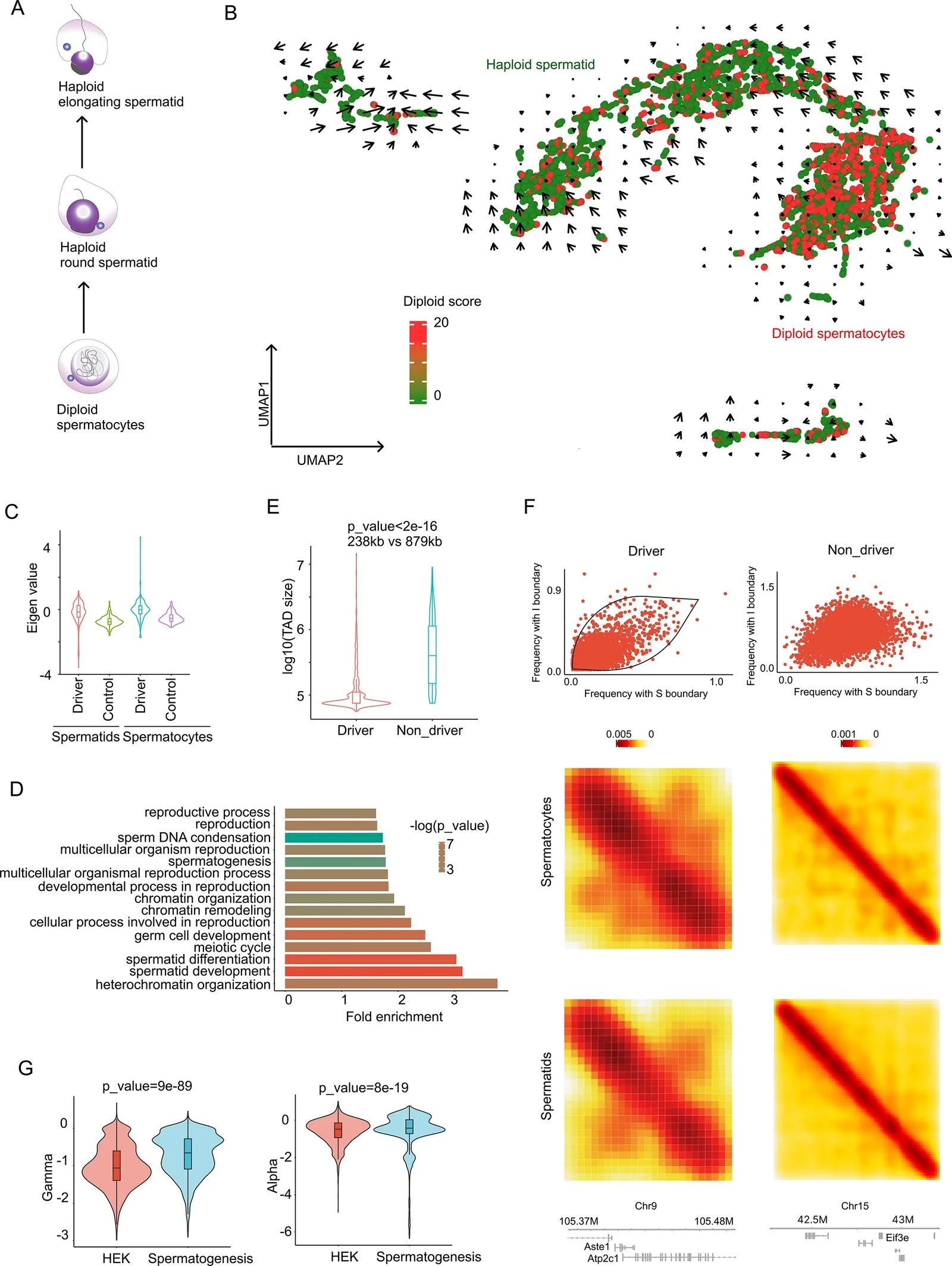
Loop velocity for spermiogenesis cells. (**A**) Illustration of the spermiogenesis process. (**B**) UMAP of spermiogenesis cells. The color gradient indicates diploid scores, 100*abs(read ratio on chrx/(chrx + chr7) - median), with higher scores being indicative of pachytene spermatocytes or earlier cells, and lower scores representing haploid spermatids. The direction of cell development is demonstrated through arrows calculated via loop velocity. (**C**) A violin plot showcasing the eigen value comparison between driving TADs and non-driving TADs. (**D**) A Gene Ontology (GO) term analysis for the top 1000 genes located within the driving TADs (based on spindle likelihood rank). (**E**) Violin plot depicting the distribution of TAD sizes between driving and non-driving TADs. (**F**) Examples of driving and non-driving TADs, showing the relationship between boundary contact distribution in each cell (dot plot), bottom contact maps (5 kb resolution) in aggregated spermatocytes and spermatids, and gene tracks. (**G**) Violin plot demonstrating the distribution of gamma and alpha values of the driving TADs in both HEK293 cells and spermiogenesis cells.

Following our established protocol, we prepared a single-cell suspension from four adult mouse testis and performed DropHiChew on these spermiogenesis cells [16]. The cell filter was configured according to the method section.

Our experiment led to the detection of around 5618 cells, each with an average of 74k contacts and a duplication rate of 67%. With a valid pair ratio of ~35.8%, the results were comparable to our earlier assessment with HEK293T.

We then utilized the UMAP embedding to map and cluster cells to evaluate the effectiveness of DropHiChew in cell-type classification and velocity prediction (Fig. 3B). Without scRNA-seq, it becomes a challenge to use structural properties for reliable cell-type classification. A key feature of meiosis is the significant change in chromosome copy number from diploid to haploid. By using the diploid scores (see “Materials and methods” section), we can confidently distinguish between diploid spermatogenic cells (such as pachytenes and earlier cells) and haploid spermatids (round and elongating spermatids). However, we could not confidently differentiate the round spermatids and elongating spermatids.

To better ascertain the accuracy of the cell types, we merged the haploid spermatids and compared the contact map with control bulk samples that were FACS-sorted [26]. We observed a similarity in eigenvalues and insulation scores, which demonstrates the high quality of our data (Supplementary Fig. S3A). Interestingly, we observed an increase in the frequency of long-distance interactions (>20 kb) in haploid spermatids (Supplementary Fig. S3B), a finding that aligns with previously published results. This could be a result of genome compaction during spermiogenesis [26].

Once we had a clear idea of the expected cell development direction, we incorporated loop velocity into our spermiogenesis data. This allowed us to determine if we could predict the development direction accurately.

### The loop velocity accurately predicts the development direction of the spermiogenesis

We have identified 2986 TADs within the spermiogenesis cells. Our research indicates that half of these TADs show an I_S plot spindle probability above 0.4, implying a significant number of suitable objects for determining loop velocity. Our analysis suggests that loop velocity, when examined using spermatogenesis data, is a reliable predictor of the expected meiosis direction, transitioning from diploid spermatogenic cells to haploid spermatids (Fig. 3B). Interestingly, prior research noted an unexpected direction when using scRNA-seq, potentially due to the short reads from scRNA underestimating the nascent RNA portion [10]. Given the strong predictability of spermiogenesis development, we performed a global quantification to assess our accuracy. The results demonstrated that loop velocity accurately predicted the expected DNA movement direction over 70% of the cases (Supplementary Fig. S4A), outperforming the velocity of scRNA-seq [10].

The process of spermiogenesis serves as an excellent model for understanding the concept of loop velocity, given the significant changes it induces in phenotype and gene expression. While RNA velocity is generally more accepted by the scientific community, this is largely because it is understood that RNA influences cell development by translating into proteins that affect phenotype. To truly grasp the biological rationale of loop velocity in cellular prediction, it is crucial to identify key TADs that drive development and investigate their functionality. It seems that the speed or direction of prediction arrows is influenced by the weight of each TAD’s predictive functions. Existing research implies that the expression of certain RNAs, especially those showing spindle-like dynamics (referred to as spindle model likelihood), may carry significant weight in these predictive functions [11]. Using similar methods, we have identified these key TADs as “driver TADs.”

We have identified ~242 “driver TADs,” with the ∼1000 gene located within them (likelihood > 0.7) (Supplementary Fig. S3C). The driver TADs exhibit a significantly higher eigenvalue than the control non-driver TADs, which suggest they may be active in transcription (Fig. 3C). Interestingly, most of the driver genes are found in the sex chromosome and play a role in spermiogenesis (Supplementary Fig. S3D). Upon closer examination, we found a 64% overlap with previously identified spermiogenesis driver genes [10] (Supplementary Fig. S3D). These genes show a distinct transcription pattern, contributing significantly to spermiogenesis progression. This strengthens the idea of the transcriptional activity of the driver TADs. Furthermore, it suggests that driver TADs might influence biological systems through their internal genes. To better understand their biological implications, we conducted a GO term analysis on the genes located within the driver TADs (Fig. 3D). The majority of these genes are involved in spermiogenesis-related processes, such as spermatid development and sperm DNA condensation. Interestingly, unlike housekeeping genes in cells that express steadily, these spermatogenesis-related genes vary in expression in different spermiogenesis phases. Overall, these findings suggest that driver TADs may enhance cellular development by promoting the expression of internal genes.

If driver TADs strongly correlate with the internal gene expression, they should fluctuate along with it, possibly driving spermatogenesis. In comparing driver and non-driver TADs, we found driver TADs to be notably smaller, with a median size of 237 kb (Fig. 3E). This aligns with expectations as larger TADs tend to hold stable structures across various cell types, as previous studies have indicated [27]. We suggest that smaller TADs might have more mobility, potentially facilitating internal gene expression. Another key observation is that around 40% of driver TADs are located in the transition region between the AB compartments. This distribution is significant compared to non-driver TAD controls, implying that these locations might offer the greatest mobility.

Upon inspecting the driver TADs of two spermatogenesis stages, we discovered significant genomic movement around the boundary, which aligns with our expectations based on spindle activity, as illustrated in the I_S plot (Fig. 3F and Supplementary Fig. S3E). Interestingly, genomic activity within non-driver TADs exhibited a random, dispersed pattern of movement. These observations lend further credence to our hypothesis that driver TADs possess greater mobility, facilitating larger internal movements.

To further globally quantify the volatility of the motion, we conducted a study comparing driver TADs influencing spermiogenesis with those affecting the HEK293T cycle. We found a significant difference (*P* < 2e-16) with a 1.3-fold change in the γ (Fig. 3G). It is important to note that γ, which is the slope of the spindle model in the I_S plot, is inversely proportional to the insulation score. Therefore, an increase in γ suggests larger and quicker shifts in genome movement. This indicates that driver TADs involved in spermatogenesis undergo more variable changes than those associated with the cell cycle. This could potentially explain why the speed arrows in the HEKs were noticeably shorter than in the spermiogenesis model, due to the smaller γ. Therefore, driver TADs are smaller and have a higher amplitude of DNA motion, which could possibly facilitate dynamic internal gene expression during spermiogenesis.

However, we still require direct evidence linking dynamic DNA movement to gene expression, which we can investigate using the multi-omics 3C data provided below.

In essence, loop velocity is key in forecasting spermatogenesis progression and pinpointing the leading TADs in chromatin remodeling. It is quite likely that these driver TADs modified internal genome structures to encourage cell differentiation by changing gene expression within the TADs.

### The loop velocity accurately predicts the development direction of the embryonic stem cells

To better understand the link between chromatin movement and gene expression, it is beneficial to have single-cell data that includes both RNA and chromatin conformation. The HiRES technology, as previously described, offers embryonic data that sequences RNA and HiC in single cells [21]. This dataset is ideal for examining the relationship between RNA velocity and loop velocity. Additionally, the embryonic stem cell data, gathered over different embryonic days, provides an opportunity to verify the precision of velocity prediction by embryonic days.

We have downloaded both RNA and HiC embryonic stem cell data for embryonic days 7.0 (E7.0) to E11.5. Around 7469 cells passed filters for both data types, yielding 2.8 billion DNA contacts. The embryonic day can be used as a time indicator to evaluate the accuracy of our predictions [21].

We implemented the loop velocity on the embryonic stem cell UMAP. Some directions align correctly, transitioning from the early to the later embryonic days (indicated by the black dash square) (Fig. 4A). However, the map’s central direction does not appear to be accurate. Upon detailed analysis, we observed that this UMAP region amalgamates both early and late embryonic cells, which are not distinctly segregated. This factor could potentially account for the diminished performance.

**Figure 4.**
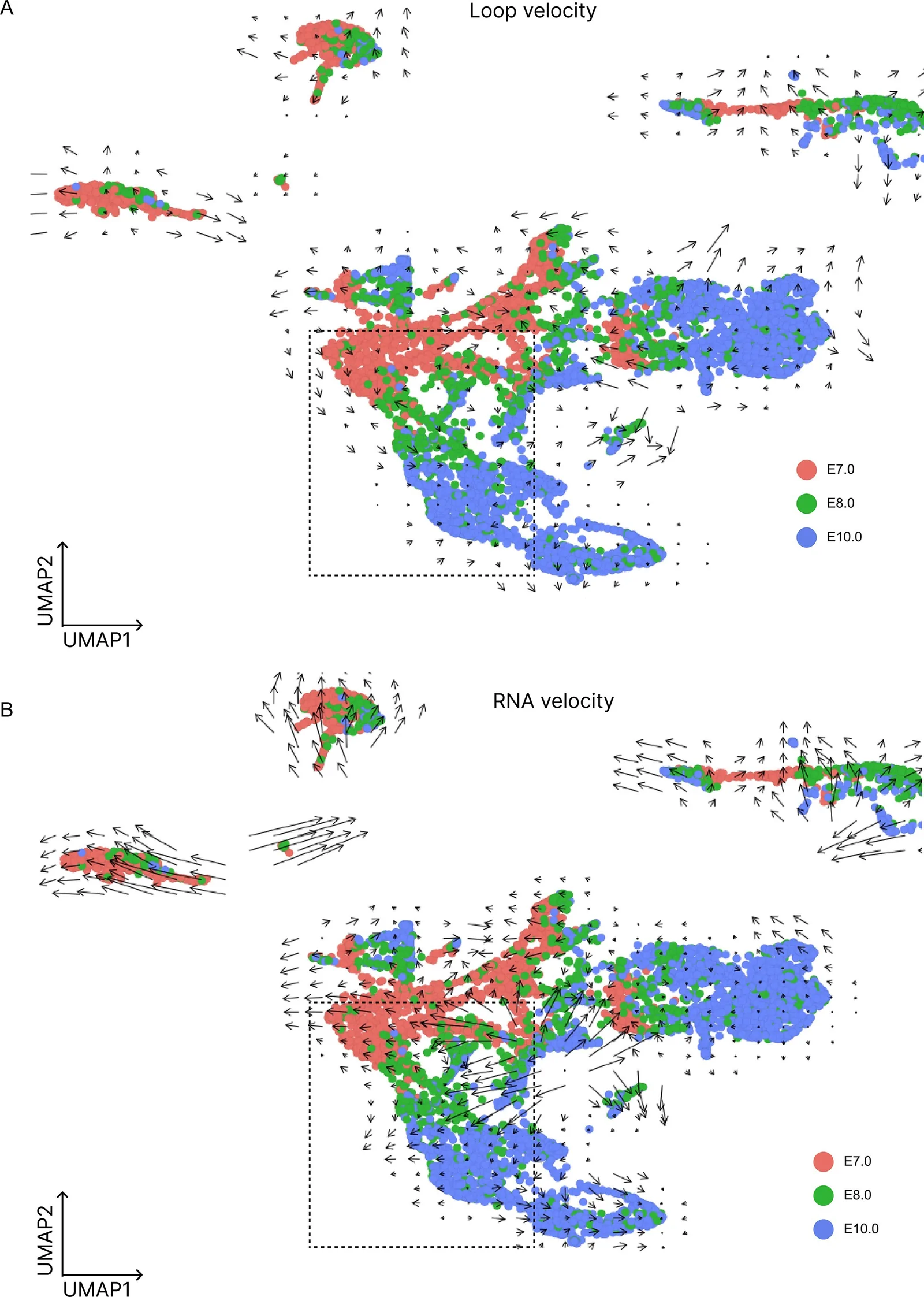
Loop velocity for embryonic cells. (**A**) A UMAP representation of embryonic cells from different stages is presented, with color gradients signifying embryonic days 7.0, 8.0, and 10.0. Arrows, derived through loop velocity calculations, indicate cell developmental direction. (**B**) The arrows’ orientation was determined using RNA velocity measurements.

We have also run the parallel RNA velocity test for comparison (see Fig. 4B). The findings suggest that RNA velocity did not perform better than loop velocity, even in areas with clear separation (indicated by the black dashed square). When we calculated the global direction in loop velocity, we found that more than half of the DNA movement predictions were heading in the same direction in the anticipated cells (Supplementary Fig. S4A).

Considering that driving TAD motion may encourage cell development via internal gene expression, we still lack direct evidence. Embryonic studies, using simultaneous RNA and 3C data from the same cell, have provided clearer insight into the potential relationship between DNA movement in TADs and gene expression. We have pinpointed contact transfer from the S boundary to the I boundary. It seems that gene expression amplifies when interactions mainly occur at the I boundary (upper curve). Conversely, when chromatin is primarily at the S boundary (lower curve), gene expression diminishes. We notice significant expression changes when the chromatin shifts between these two boundaries (Supplementary Fig. S4C). Further analysis shows that this pattern is consistent in ~92% of TADs, suggesting a strong correlation between gene expression and chromatin movement.

In summary, we have introduced the concept of loop velocity through the simple experimental system of DropHiChew. This novel approach allows us to introduce the dynamic parameters α and γ to describe the dynamic movement of the TAD and predict the direction of cellular development. Our loop velocity model has shown high predictive accuracy across various cell types, including HEK293T cells, spermatogenesis cells, and embryonic stem cells. Intriguingly, our findings suggest that loop velocity not only predicts cellular development direction but also identifies driver TADs that influence this development through changing the gene expression within TADs. This research provides valuable insights into the dynamics of chromatin architecture within individual cells, offering a valuable tool for future studies in cellular development.

## Discussion

While droplet-based single-cell Hi-C methods have recently emerged [7], existing approaches still face limitations in data efficiency due to barcode-GATC interference. To address this, we developed DropHiChew, a method ​adapted for​ commercial droplet-based platforms to enable robust single-cell 3C profiling. Our unique approach to post-PCR enrichment technology significantly improves data usage, achieving four times the performance of non-enrichment method and making it more accessible to general labs. Simultaneously, we introduce the concept of loop velocity, an innovative method to predict cellular development direction by examining the dynamics of TADs. With its accuracy in predicting cellular development direction, loop velocity also helped us identify key TADs driving development, providing fresh insights into the dynamics of chromatin architecture within individual cells.

We need to address a critical shortfall in our 3C experiment—given the use of Tn5, it is natural to question whether Tn5 might create a bias toward accessible regions. However, after comparing the HiChew (open/close ratio 0.1), a ligation-based method, and DropHiChew (open/close ratio 0.02), a Tn5-based method, we did not observe the contact bias toward accessible regions. In fact, we found that Tn5 exhibits less preference for the accessible region in the DropHiChew. This is likely due to heated SDS being able to resolve most compact chromatins. The similarity of the contact maps further supports this point of absence of bias toward accessible chromatin. However, there are differences that exist. We believe that the limitations of Tn5 should be given more consideration in future 3C research.

The other shortage in these experiments, we used the DpnII-*dam* methyltransferases to perform the DropHiChew. Therefore, the digestion enzyme selection needs the corresponding the methyltransferase to labeling the ligation scar in order to perform the post PCR enrichment. This combination selection is around 20 different enzymes, which might limit your choice of enzyme. Moreover, multiple enzyme digestion is not available for this method. Therefore, the high-resolution maps are not achievable in the DropHiChew.

Despite being designed to address the GATC barcode bias in immunoprecipitation, the DropHiChew still exhibits some residual bias. We suspect this could be due to partial primer extension causing false priming in certain locations, particularly in the barcode regions. This can lead to the generation of double-stranded barcode DNA that may be labeled in subsequent steps. If your analysis is sensitive to this, we recommend excluding ~5% of your data that includes the GATC barcode in downstream analysis. However, we have not observed any significant issues in our loop velocity analysis.

In the context of loop velocity applications, we discovered that the key to accurate results lies in UMAP clustering. The inference of loop velocity is inherently sensitive to the resolution of cell state separation achieved in these embeddings. However, both our work and previous studies have demonstrated that 3C-based data typically offer lower discriminative power than RNA-seq at the single-cell level [21–23], due in part to their sparsity and the challenge of capturing subtle conformational changes in a reduced-dimensional space. When 3C data fail to capture fine-grained cellular variations in low-dimensional embeddings such as UMAP, it may compromise the accuracy of loop velocity inference—even though our method performs reasonably well under suboptimal clustering conditions. This underscores the need for more advanced clustering approaches specifically tailored to chromatin conformation data. Promising directions include the development of dimensionality reduction and clustering algorithms that directly leverage the graph-structured nature of chromatin contacts, or that can jointly model multi-omics data streams to construct a unified, higher-fidelity cell state manifold. Encouragingly, several recently developed co-assay technologies now allow simultaneous profiling of RNA expression and 3D genome architecture in the same cell. Alternatively, integrative analysis of multi-omics datasets can enhance cell state resolution [28], thereby providing a more reliable basis for loop velocity analysis. Furthermore, since DropHiChew is designed around the 10× Genomics framework, it can be readily adapted to platforms such as the Chromium Single Cell Multiome ATAC + Gene Expression system, enabling truly simultaneous measurement of transcriptome and chromatin conformation in individual cells and offering a direct experimental path to overcome the current embedding challenge.

Promising directions for improving clustering quality include the development of dimensionality reduction algorithms that directly leverage the graph-structured nature of chromatin contacts, such as graph neural networks or topology-aware embedding methods. Additionally, joint modeling of multi-omics data streams—combining chromatin conformation with transcriptomic or epigenetic information—can construct a unified, higher-fidelity cell state manifold. Encouragingly, co-assay technologies like HiRES [21] and scCARE-seq [22] now enable simultaneous profiling of RNA expression and 3D genome architecture in the same cell. Since DropHiChew is designed around the 10X Genomics framework, it can be readily adapted to platforms such as the Chromium Single Cell Multiome ATAC + Gene Expression system, enabling truly simultaneous measurement of transcriptome and chromatin conformation and offering a direct experimental path to overcome the current embedding challenge.

The interplay between genome folding in cell cycles and cell differentiation can be intricate, yet it is essential, particularly in embryonic cells where these processes are happening at the same time. We are primarily exploring whether cell differentiation or the cell cycle is more influential in our predictions. As per our driver TAD analysis, TADs associated with cell differentiation appear to hold the most predictive value. This could be attributed to these TADs typically having higher γ factors than those linked to the cell cycle (Fig. 3G). So, in our research, it seems that TADs related to cell differentiation are the key determinants of prediction direction. Additionally, we predict local speed using local neighboring data points on the UMAP. If the two kinds of driver TADs don’t vary simultaneously, they shouldn’t interfere with each other in the analysis.

In the loop velocity theory, we mainly discussed one-sided extrusion models. Yet, some scientists might suggest that TAD forms from both sides [29]. To address this, we have also developed a two-sided extrusion model, which you’ll find in the supplemental notes for further exploration. We used statistical tests to examine the contact density on both sides to determine if these TADs resulted from one or two-sided extrusion. Interestingly, we found that 70% of TADs rely on one-sided extrusion, with the remainder depending on two-sided extrusion. The one-sided model could cover most of the TADs in our downstream analysis.

The application of dynamical models to single-cell 3C data presents distinct computational challenges that differ from those in RNA velocity. Unlike transcriptomic data, chromatin contact matrices are inherently sparse and high-dimensional, necessitating robust parameter estimation methods. Our loop velocity framework, which relies on quantile regression, provides a stable foundation for inferring chromatin dynamics from these sparse observations. Looking forward, the field will benefit from the development of algorithms specifically designed to handle the noise characteristics of chromatin conformation data. Furthermore, the integration of loop velocity with multi-omics modalities, such as simultaneous RNA-3C measurements, will demand novel computational approaches to jointly model transcriptional and structural dynamics, offering a more unified view of cellular fate transitions.

In general, this study establishes ​DropHiChew​ as a highly efficient, commercially accessible platform for single-cell 3D genome mapping, leveraging droplet microfluidics and targeted ligation-scar enrichment to achieve superior data utility (30.3% valid pair ratio). Integrated with our ​loop velocity​ algorithm—a dynamic model quantifying TAD formation (α) and dissolution (γ) rates from chromatin contact patterns—we demonstrate robust prediction of cellular trajectories (>70% directional accuracy) across diverse biological contexts, including spermiogenesis and embryonic development. Critically, loop velocity identifies compact ​driver TADs​ (median 237 kb) enriched at AB compartment boundaries, whose enhanced structural dynamics spatially coordinate developmental gene expression programs. This unified experimental-computational framework provides a foundational approach to dissect the kinetic principles governing genome architecture and its functional coupling to cell fate transitions.

## Supplementary Material

gkag995_Supplemental_Files

## Data Availability

You can find the data for this study at the following locations: NCBI BioProject PRJNA1127783. We also used other public datasets from NCBI GEO, which you can access using these identifiers: ChIP-seq (CTCF ENCSR135CRI, H3K4me3 ENCSR000DTU; and H3K27ac ENCSR000FCH), snHi-C (GSE94489), Dip-C (GSE146397), HEK293T *in situ* Hi-C 2 (GSE143465), SCA-seq (PRJNA917827). The source data come with this paper. Custom scripts used in this study are available from https://github.com/genometube/DropHiChew, and the R package of loop velocity is available from https://github.com/Dekayzc/Loopvelocity. These are also available on Figshare at https://figshare.com/articles/software/Loopvelocity/33366150?file=67949058.
