# Supplementary material for: Exploring DNA movement through the application of droplet-based high-efficiency chromatin conformation capture (DropHiChew) and loop velocity
Source: Nucleic Acids Res. 2026 Oct 29;54(20):gkag995. doi: 10.1093/nar/gkag995 (PMC13717199; doi:10.1093/nar/gkag995)
Supplement: Supplementary file 1 [file gkag995_supplemental_files.zip › Supplement figures and legend.pdf]

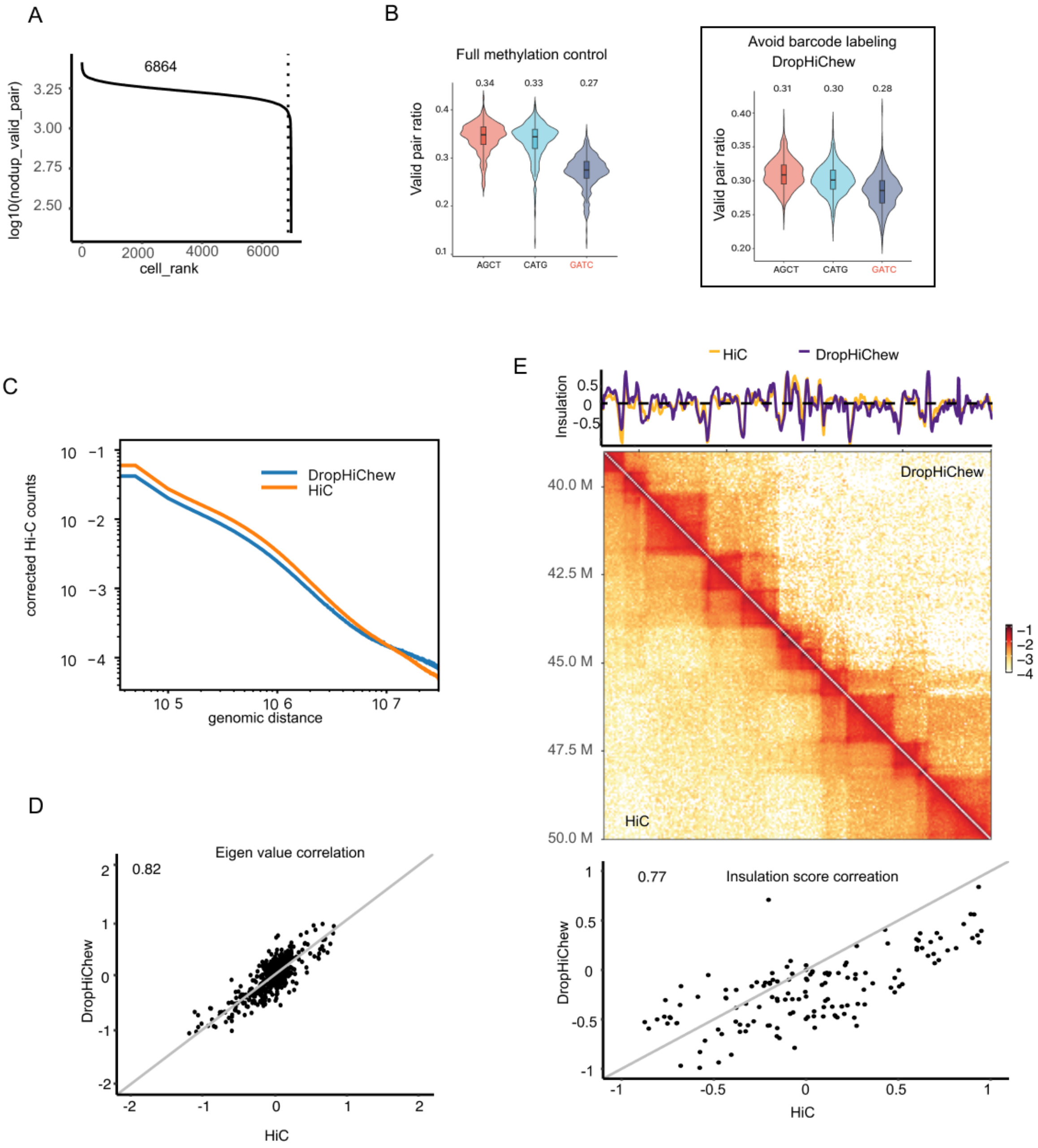

A
6864
log10(nodup_valid_pair)
cell_rank
B
Full methylation control
Valid pair ratio
0.34
0.33
0.27
AGCT
CATG
GATC
Avoid barcode labeling
DropHiChew
0.31
0.30
0.28
C
corrected Hi-C counts
DropHiChew
HiC
genomic distance
D
Eigen value correlation
0.82
DropHiChew
HiC
E
HiC
DropHiChew
Insulation
40.0 M
42.5 M
45.0 M
47.5 M
50.0 M
0.77
Insulation score correation

Supplemental Figure 1:(A) Line plot representation of the cell rank curve, focusing on deduplicated valid contacts, providing an overview of cell-to-cell variability in chromatin conformation capture studies. (B) Violin plot illustrating the valid report ratio (valid pairs to valid reads) for both control (featuring fully labelled barcodes) and DropHiChew (which omits barcode labelling). Control barcodes with AGCT or CATG motifs are denoted as AGCT CATG, while GATC represents barcodes with a GATC motif. (C) Line plot visualizing the contact distance decay curve of DropHChew, offering insights into the decline rate of contact frequency correlating with increased genomic distance. (D) Dot plot detailing the correlation of eigenvalues between DropHChew and HiC, showcasing the consistency between these two methods in capturing chromatin interaction patterns. (E) Comparative analysis of contact maps at a 50kb resolution, insulation score distribution, and insulation score correlation between HiC and DropHChew, highlighting the similarities and differences in chromatin architecture as captured by these two methods.

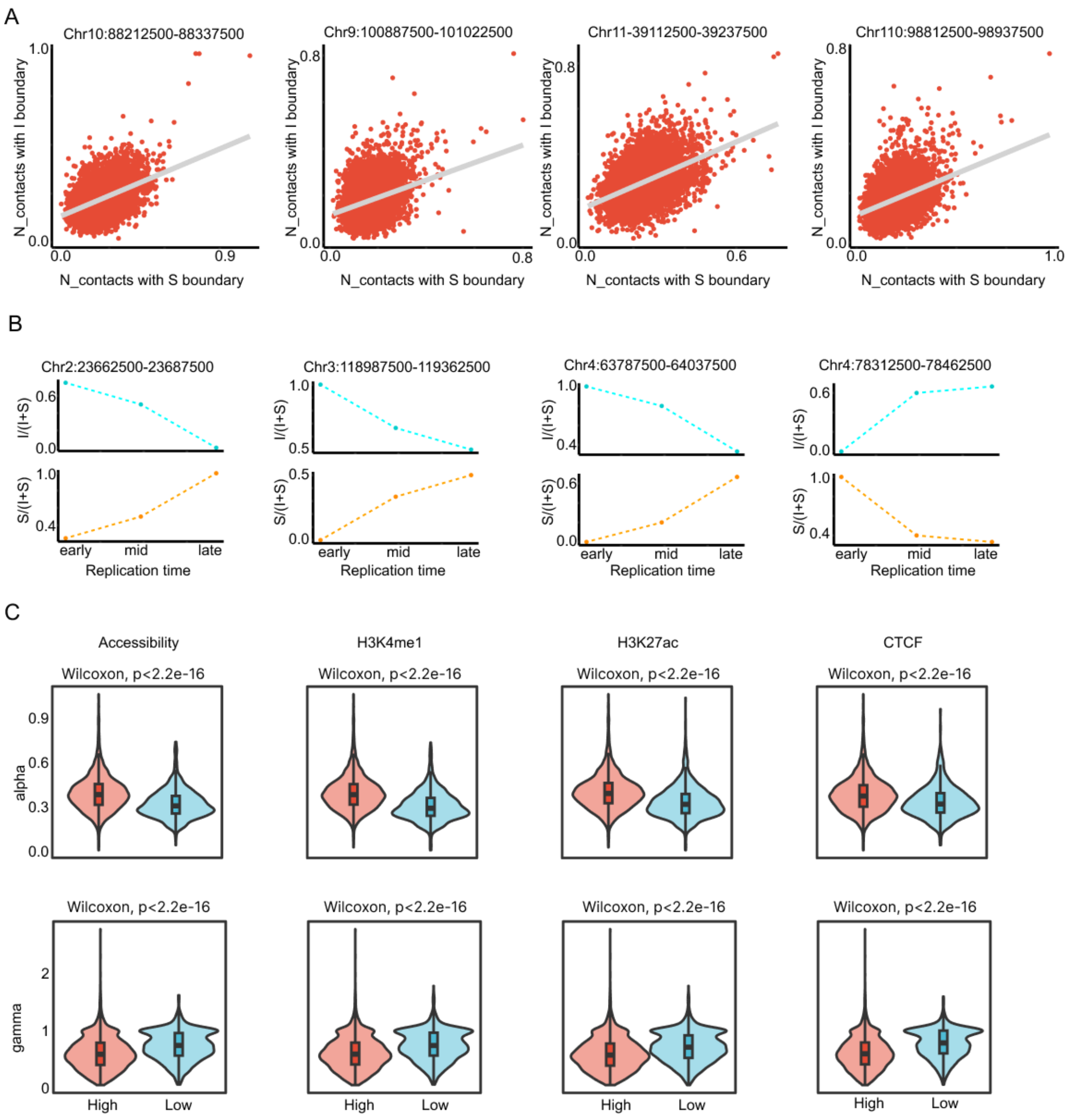
A
Chr10:88212500-88337500
Chr9:100887500-101022500
Chr11-39112500-39237500
Chr110:98812500-98937500
N_contacts with I boundary
N_contacts with S boundary
B
Chr2:23662500-23687500
Chr3:118987500-119362500
Chr4:63787500-64037500
Chr4:78312500-78462500
I/(I+S)
S/(I+S)
early
mid
late
Replication time
C
Accessibility
H3K4me1
H3K27ac
CTCF
Wilcoxon, p<2.2e-16
alpha
gamma
High
Low

Supplemental Figure 2: (A) This line plot illustrates the average boundary contact ratio within an example TAD for both I boundary and S boundary (HEK293). (B) The dot plot portrays the distribution of normalized interactions between the I boundary and S boundary in individual cells within example regions (HEK293). (C) The violin plots at the top and bottom reveal the alpha and gamma distributions across high (upper quantile) and low (lower quantile) chromatin accessibility, H3K4me1, H3K27ac, and CTCF (HEK293).

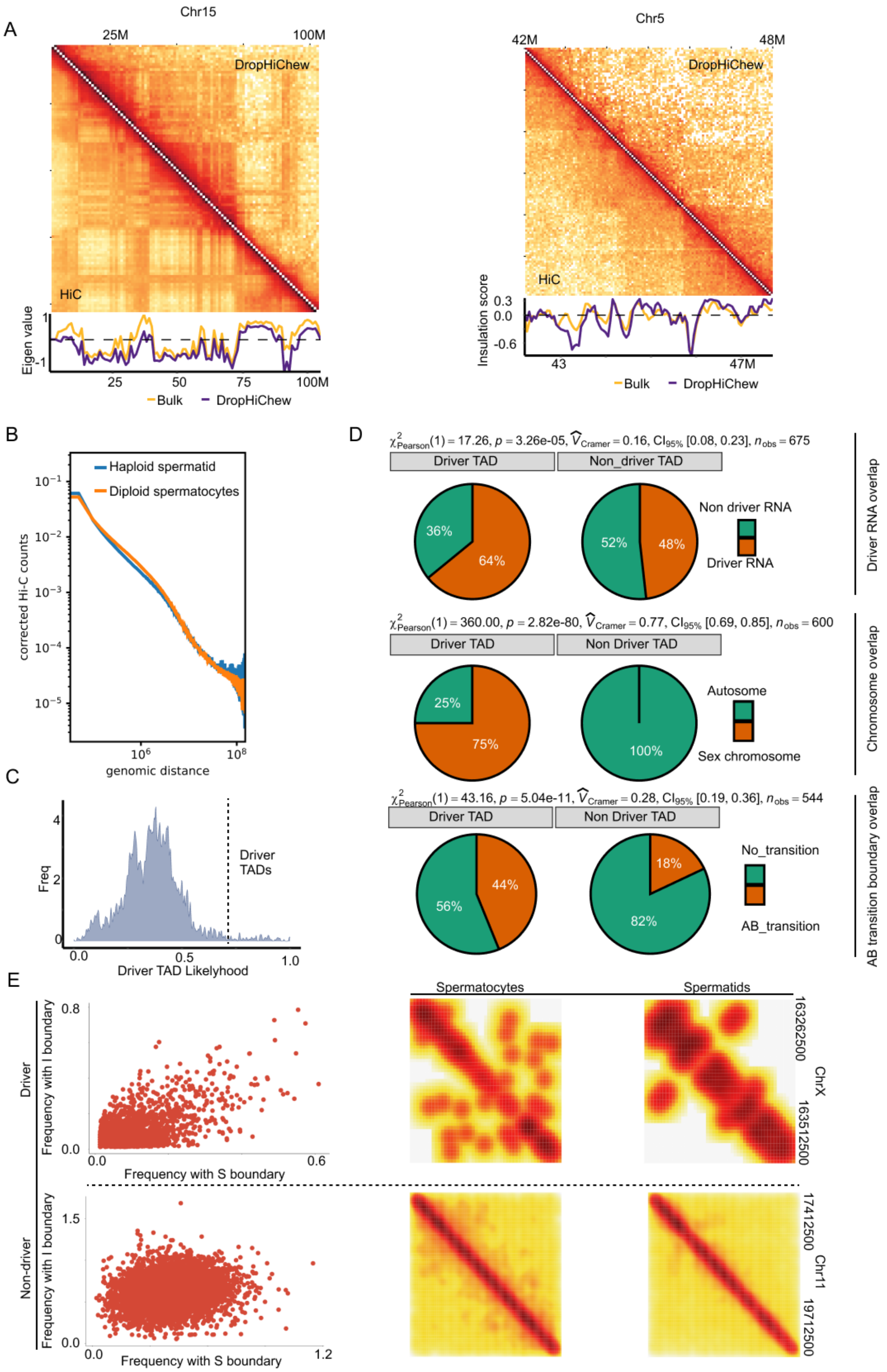
A
Chr15
25M
100M
DropHiChew
HiC
Eigen value
Bulk
DropHiChew
Chr5
42M
48M
Insulation score
43
47M
B
Haploid spermatid
Diploid spermatocytes
corrected Hi-C counts
genomic distance
C
Freq
Driver TADs
Driver TAD Likelihood
D
Driver TAD
Non_driver TAD
Non driver RNA
Driver RNA
Driver RNA overlap
Non Driver TAD
Autosome
Sex chromosome
Chromosome overlap
No_transition
AB_transition
AB transition boundary overlap
E
Driver
Frequency with I boundary
Frequency with S boundary
Non-driver
Spermatocytes
Spermatids
ChrX
163262500
163512500
Chr11
17412500
19712500

Supplemental Figure 3: (A) Comparative contact maps for DropHiChew and HiC at a resolution of 1Mb are shown at the top (haploid spermatids). The lower panel displays the eigenvalue distribution across the genome for both the techniques. On the right, a set of plots provide a 50kb resolution and insulation score distribution. (B) The graph depicts the decay curve of contact distance in haploid spermatids and diploid spermatocytes. (C) The density plot illustrates the distribution of spindle shape likelihood for TADs, with TADs above 0.7 being classified as driver TADs in spermiogenesis. (D) The pie chart displays the Chi-square test distribution of the overlap between driver/non-driver TADs and driver/non-driver genes in spermiogenesis. The middle pie chart shows the distribution preference of driver/non-driver TADs between the autosome and sex chromosome. The bottom pie chart indicates the preferential distribution of driver/non-driver TADs on the AB transition domain, defined as a 1MB region across the AB compartments. (E) Contact maps for example regions of driver and non-driver TADs, such as ChrX:163262500-163512500 (driver TAD) and Chr11:17412500-19712500 (non-driver TAD), are presented. The distribution of normalized contacts between the Initiation (I) and Stabilization (S) boundaries across all cells is illustrated in the left dot plot.

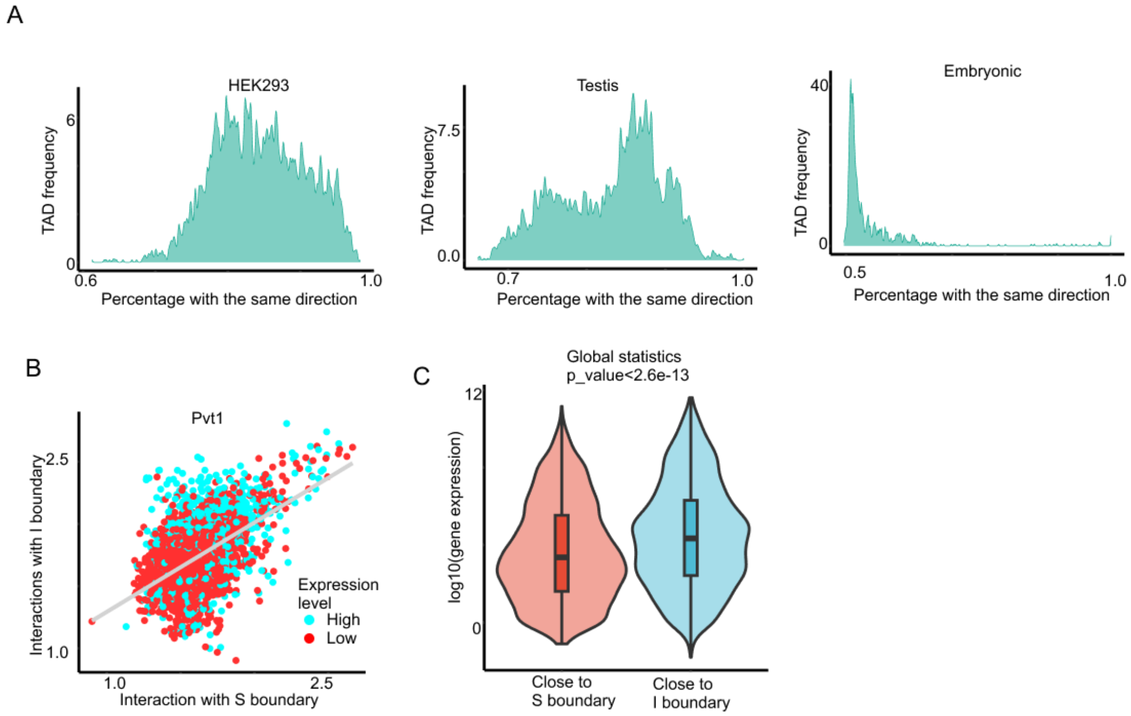

A
HEK293
TAD frequency
6
0
0.6
1.0
Percentage with the same direction
Testis
TAD frequency
7.5
0.0
0.7
1.0
Percentage with the same direction
Embryonic
40
TAD frequency
0
0.5
1.0
Percentage with the same direction
B
Pvt1
Interactions with I boundary
2.5
1.0
1.0
2.5
Interaction with S boundary
Expression level
High
Low
C
Global statistics
p_value<2.6e-13
12
0
log10(gene expression)
Close to S boundary
Close to I boundary

Supplemental Figure 4: (A) A density plot displaying the distribution of the correct prediction percentage for Topologically Associating Domains (TADs) across all cells. The correct prediction refers to instances where the anticipated direction of DNA movement matches the actual subsequent movement. The x-axis represents the correct prediction percentage for all events within each TAD. (B) A dot plot visualizing the interaction frequency distribution between the initiation(I) and stabilization (S) boundaries in embryonic cells. High expression of Pvt1 (top half) is denoted in blue, and low expression (bottom half) in red. (C) A violin plot representing the overall gene expression trends as genes approach the I and S boundaries within embryonic cells.

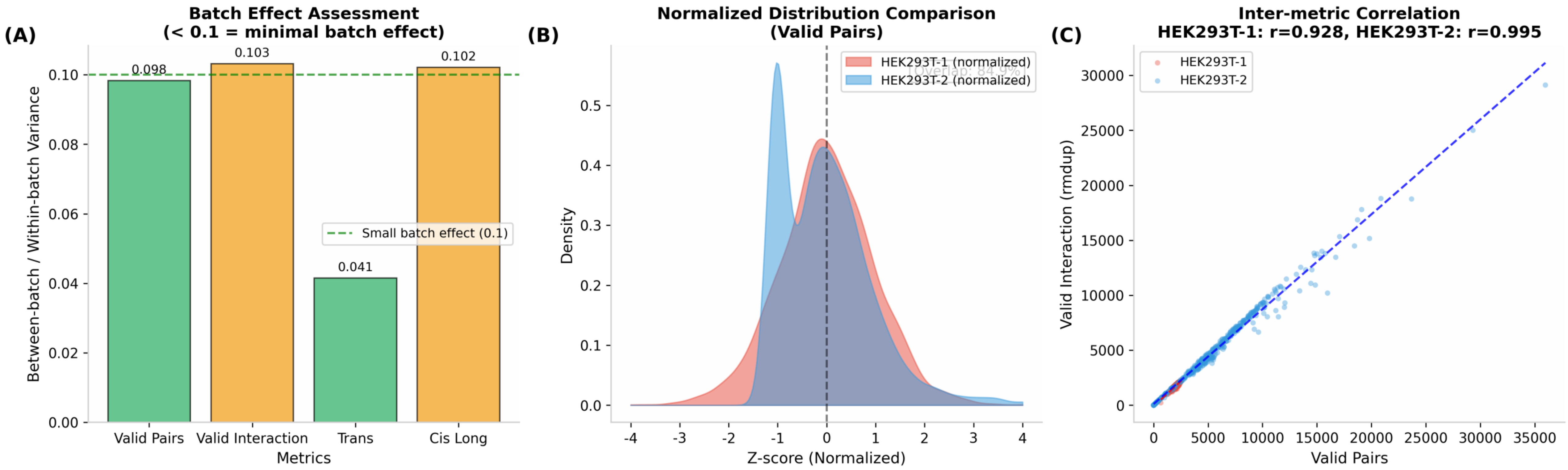
(A)
Batch Effect Assessment
(< 0.1 = minimal batch effect)
Between-batch / Within-batch Variance
0.098
0.103
0.041
0.102
Small batch effect (0.1)
Valid Pairs
Valid Interaction
Trans
Cis Long
Metrics
(B)
Normalized Distribution Comparison
(Valid Pairs)
HEK293T-1 (normalized)
HEK293T-2 (normalized)
Density
Z-score (Normalized)
(C)
Inter-metric Correlation
HEK293T-1: r=0.928, HEK293T-2: r=0.995
HEK293T-1
HEK293T-2
Valid Interaction (rmdup)
Valid Pairs

Supplementary Figure 5: (A) Batch effect assessment showing the ratio of between-batch variance to within-batch variance between HEK293T-1 and HEK293T-2 replicates. All ratios are < 0.11 (Valid Pairs: 0.098, Valid Interaction: 0.103, Trans: 0.041, Cis Long: 0.102), with three metrics below the threshold of 0.1 for minimal batch effect. The green dashed line indicates the threshold for small batch effect (0.1). (B) Normalized distribution comparison (Z-score) of Valid Pairs between HEK293T-1 (red) and HEK293T-2 (blue). The high overlap coefficient (84.9%) indicates that after normalization, the two replicates follow highly similar distributions. (C) Inter-metric correlation showing the relationship between Valid Pairs and Valid Interaction (rmdup) within each replicate. Both replicates show strong correlations (HEK293T-1: r = 0.928, HEK293T-2: r = 0.995), indicating consistent quality metric relationships. Red and blue dashed lines represent linear regression fits for HEK293T-1 and HEK293T-2, respectively. These analyses demonstrate minimal batch effect between HEK293T-1 and HEK293T-2 replicates. The between-batch variance is substantially smaller than within-batch variance for all metrics, confirming high reproducibility of the DropHiChew method.

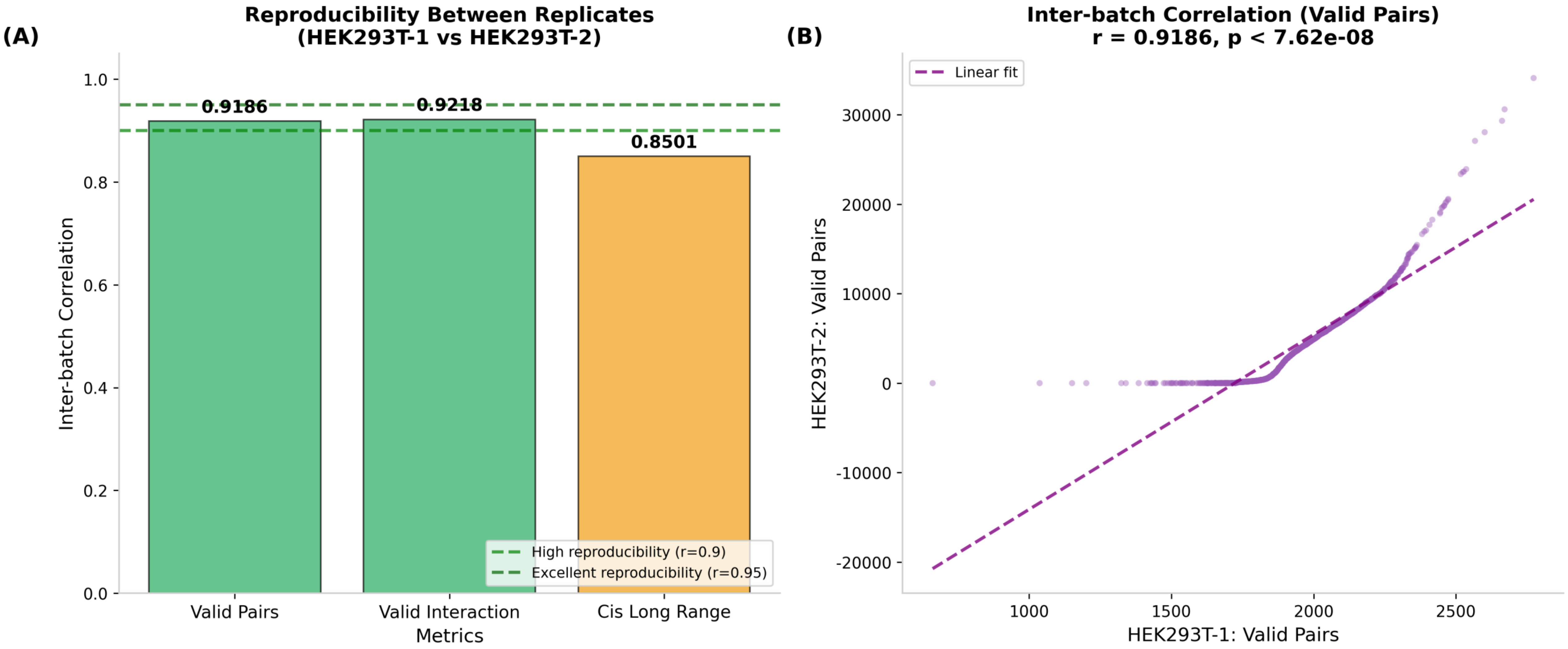
(A)
Reproducibility Between Replicates
(HEK293T-1 vs HEK293T-2)
Inter-batch Correlation
1.0
0.8
0.6
0.4
0.2
0.0
0.9186
0.9218
0.8501
High reproducibility (r=0.9)
Excellent reproducibility (r=0.95)
Valid Pairs
Valid Interaction Metrics
Cis Long Range
(B)
Inter-batch Correlation (Valid Pairs)
r = 0.9186, p < 7.62e-08
Linear fit
HEK293T-2: Valid Pairs
30000
20000
10000
0
-10000
-20000
1000
1500
2000
2500
HEK293T-1: Valid Pairs

Supplementary Figure 6: (A) Inter-batch correlation between HEK293T-1 and HEK293T-2 replicates for key Hi-C quality metrics. Valid Pairs ($r = 0.9186$), Valid Interaction ($r = 0.9218$), and Cis Long Range ($r = 0.8501$) all show high correlations (> 0.85), demonstrating excellent reproducibility between replicates. The green dashed lines indicate thresholds for high ($r = 0.9$) and excellent ($r = 0.95$) reproducibility. (B) Scatter plot showing the correlation of Valid Pairs between HEK293T-1 and HEK293T-2 replicates ($r = 0.9186$, $p < 1e-6$). Each point represents a cell, with cells ranked by Valid Pairs in each replicate. The purple dashed line represents the linear regression fit. The strong linear relationship demonstrates high reproducibility of the DropHiChew method across replicate experiments. These analyses demonstrate excellent reproducibility of the DropHiChew method. The high inter-batch correlation coefficients (> 0.85) confirm that quality metrics are highly consistent between replicate experiments.

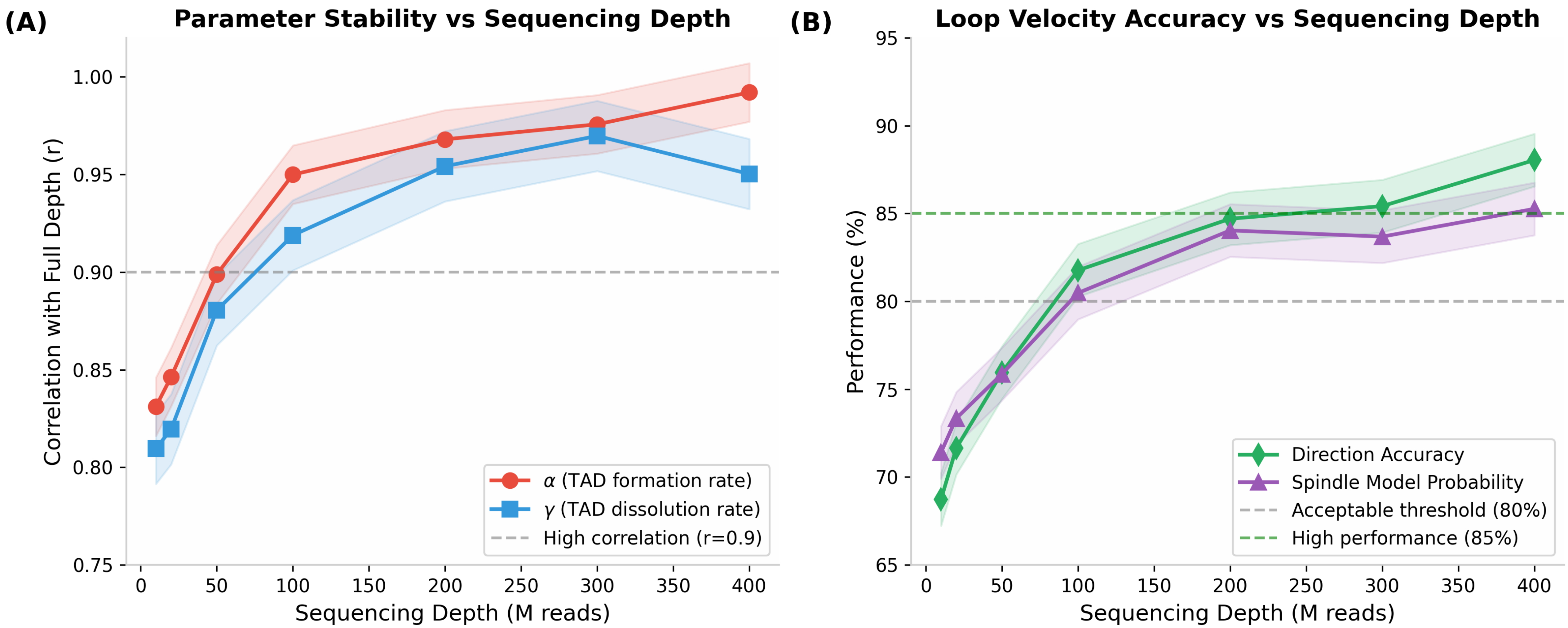
(A)
Parameter Stability vs Sequencing Depth
Correlation with Full Depth (r)
Sequencing Depth (M reads)
α (TAD formation rate)
γ (TAD dissolution rate)
High correlation (r=0.9)
(B)
Loop Velocity Accuracy vs Sequencing Depth
Performance (%)
Sequencing Depth (M reads)
Direction Accuracy
Spindle Model Probability
Acceptable threshold (80%)
High performance (85%)

Supplementary Figure 7: (A) Parameter stability analysis showing the correlation of alpha (TAD formation rate, red circles) and gamma (TAD dissolution rate, blue squares) parameters with full-depth (400M reads) values across different sequencing depths. Both parameters show high stability (r > 0.9) at sequencing depths >=100M reads (alpha: r = 0.950, gamma: r = 0.919 at 100M). The gray dashed line indicates the threshold for high correlation (r = 0.9). (B) Loop velocity accuracy analysis as a function of sequencing depth. Direction accuracy (green diamonds) represents the percentage of correctly predicted TAD movement directions, while spindle model probability (purple triangles) indicates the likelihood of TADs conforming to the expected spindle-shaped distribution. Both metrics exceed 80% at 100M reads and reach >85% at >=200M reads. The gray and green dashed lines indicate acceptable (80%) and high performance (85%) thresholds, respectively. These analyses demonstrate that loop velocity calculations are robust to sequencing depth variations. Results at 100M reads are highly consistent with 400M reads (alpha: r = 0.950, gamma: r = 0.919, direction accuracy: 81.8%, spindle probability: 80.5%), indicating that moderate sequencing depths are sufficient for accurate loop velocity inference.
