# Supplementary material for: Exploring DNA movement through the application of droplet-based high-efficiency chromatin conformation capture (DropHiChew) and loop velocity
Source: Nucleic Acids Res. 2026 Oct 29;54(20):gkag995. doi: 10.1093/nar/gkag995 (PMC13717199; doi:10.1093/nar/gkag995)
Supplement: Supplementary file 1 [file gkag995_supplemental_files.zip › Supplementary_Notes.docx]

**Supplementary notes: Mathematical inference and computational methods.**

**Inference of Loop velocity**

Developing a kinetic model for DNA could unlock the potential for time series analysis akin to that of RNA, where the concept of RNA velocity allows for the extrapolation of cellular time series from RNA kinetics. In this study, we choose loop extrusion model as DNA kinetic model. Hi-C sequencing has revealed a wealth of spatial structures within DNA. By analyzing the Hi-C interaction matrix, we can discern various organizational features such as chromosome territories, A/B compartments, topologically associating domains (TADs), and chromatin loops. Our study will use parameters and features in TADs.

Loop extrusion can occur in two modes: one-sided or two-sided. In the one-sided mode, the regions in initiation boundary (I boundary) remains stationary while the other regions in stabilizing boundary (S boundary) oscillates, generating a loop structure. We hypothesize that the frequency of contact formation per unit time during loop extrusion is a constant, *α*. As the S boundary undergoes oscillation, it not only forms contacts with other regions but also experiences a reduction in contacts. At the I boundary, the decrease in contacts at the S boundary corresponds to an increase in contacts at the I boundary. When loop structure is dissociated, the contacts in I boundary will be decreased. From this one-sided loop extrusion process, we can derive the following ordinary differential equation (ODE):

$$\frac{dS}{dt}=\alpha-\beta S\left( t \right), \left( 1 \right)$$

$$\frac{dI}{dt}=\beta S\left( t \right)-\gamma I\left( t \right), \left( 2 \right)$$

Here, *S(t)* and *I(t)* correspond to the contacts in about 50kb regions around S and I boundaries respectively in specific time *t*. *β* represents the rate of active contact reduction at the S boundary during loop formation, while *γ* signifies the rate of loop dissociation at the I boundary. The analytical solution to the first order linear differential equations (Eq. 1 and 2) can be

$$S\left( t \right)=\frac{\alpha}{\beta}+(S_{0}-\frac{\alpha}{\beta})e^{-\beta t}, \left( 3 \right)$$

$$I\left( t \right)=\frac{\alpha}{\gamma}+\left( I_{0}-\frac{\alpha}{\gamma} \right)e^{-\gamma t}+\frac{\alpha-\beta S_{0}}{\gamma-\beta}(e^{-\gamma t}-e^{-\beta t}) \left( 4 \right)$$

Where *I_0_*=*I*(0), *S_0_*=*S*(0). Normally, we can assume *I_0_*=0, *S_0_*=0. Then, the analytical solution can be

$$S\left( t \right)=\frac{\alpha}{\beta}-\frac{\alpha}{\beta}e^{-\beta t}, \left( 5 \right)$$

$$I\left( t \right)=\frac{\alpha}{\gamma}-\frac{\alpha}{\gamma}e^{-\gamma t}+\frac{\alpha}{\gamma-\beta}(e^{-\gamma t}-e^{-\beta t}) \left( 6 \right)$$

In a two-sided loop extrusion scenario, the S boundary behaves similar as that in one-sided scenario, while the I boundary behaves similarly to the S boundary, with the DNA molecule translocating through DNA forth and back within the confines of the SMC complex loop, forming a loop in conjunction with the S boundary. Consequently, the overall rate of contact formation α and the rate of loop dissociation *γ* attributed to the loop extrusion process is divided between the S and I boundaries by ratio *θ*. Both boundaries experience an increase in contact numbers due to their proximity, as well as a decrease in contacts as dissociation of loop structure. From the two-sided loop extrusion mechanism, we can derive the following ODE:

$$\frac{dS}{dt}=\theta\alpha-\theta\gamma S(t), \left( 7 \right)$$

$$\frac{dI}{dt}=\left( 1-\theta\right)\alpha-(1-\theta)\gamma I\left( t \right), \left( 8 \right)$$

The analytical solution can be

$$S\left( t \right)=\frac{\alpha}{\gamma}+(S_{0}-\frac{\alpha}{\gamma})e^{-\theta\gamma t}, \left( 9 \right)$$

$$I\left( t \right)=\frac{\alpha}{\gamma}+(I_{0}-\frac{\alpha}{\gamma})e^{-(1-\theta)\gamma t}, \left( 10 \right)$$

Where *I_0_*=*I* (0), *S_0_*=*S* (0). Normally, we can assume *I_0_*=0, *S_0_*=0. Then, the analytical solution can be

$$S\left( t \right)=\frac{\alpha}{\gamma}-\frac{\alpha}{\gamma}e^{-\theta\gamma t}, \left( 11 \right)$$

$$I\left( t \right)=\frac{\alpha}{\gamma}-\frac{\alpha}{\gamma}e^{-(1-\theta)\gamma t}, \left( 12 \right)$$

Determining whether the loop extrusion process is one-sided or two-sided can be achieved by analyzing the number of contacts at the TAD boundaries in each cell within a single-cell Hi-C bulk sample. A t-test can be employed to assess the significance of the difference in contact numbers at both boundaries. If no significant difference is detected, we assume the loop extrusion process is one-sided. Conversely, if a significant difference is observed, we assume the loop extrusion process is two-sided with the boundary exhibiting a higher number of contacts designated as the S boundary, and the opposite boundary as the I boundary.

Therefore, the parameters that have to be inferred are *φ* (*α*, *β*, *γ*) for one-sided loop extrusion model and *φ* (*α*, *θ*, *γ*) for two-sided loop extrusion model. Next, we will elaborate how these parameters are estimated.

**Inference of parameters and extrapolation of cell state**

**Steady-state model**

To resolve the unknown parameters *φ* (*α*, *β*, *γ*) for one-sided loop extrusion model, we can infer their values in a steady state, where *dI* / *dt* equals zero. By applying this steady state, the value of γ can be estimated through linear regression. This approach to parameter estimation is analogous to that used in RNA velocity. However, it is crucial to understand the biological significance of the steady state. A steady state at boundary I, where *dI* / *dt* is zero, implies a relative constancy in the number of contacts, with increases offset by decreases. In the context of the loop extrusion model, this suggests that DNA molecules oscillate at both boundaries S and I, as evidenced by the work of the Mirny Lab. Their visual demonstrations have provided insights into how DNA maintains a certain equilibrium, given that DNA is theoretically in perpetual motion.

In steady state, when *dI* / *dt* = 0, equation 2 would be

$$\beta S\left( t \right)-\gamma I\left( t \right)=0, then S\left( t \right)=\frac{\gamma}{\beta}I(t) \left( 13 \right)$$

Here, we set β = 1 as a reference, effectively normalizing the other parameters (α, γ) relative to β. Consequently, the absolute values of α and γ are defined on this relative scale. If β is assigned a different value, the inferred values of α and γ would change proportionally, as the entire system of parameters is defined up to a multiplicative constant. This rescaling does not alter the model's core predictions, which depend on the ratios between parameters (e.g., α/γ), rather than their absolute magnitudes. Then, linear fit model using a least square fit of *S* and *I* from observed data can solve the parameter *γ* using same inference process in RNA velocity as the linear fit equations are the same.

For two-sided loop extrusion model, the steady state is not same as one-sided loop extrusion model. The balance between *S* and *I* boundary occur when velocities of both boundary reached the same. When *dS* / *dt* = *dI* / *dt*, combined with equation 7 and 8, the solution would be

$$S\left( t \right)=\frac{1-\theta}{\theta}I\left( t \right)+\frac{\alpha}{\gamma}(2-\frac{1}{\theta}) \left( 14 \right)$$

Let *α*=1, which means the generation rate of TADs in DNA is unit rate 1. The parameters *θ* and *γ* could be solved using linear fit model based on a least square fit of *S* and *I* from observed data. Current cell state could be represented by the observed value *I* of all TADs. So, projected cell state could be represented by the extrapolated value *I* of all TADs. For a single TAD in each cell, the observed value *I* can be regarded as *I*(t) in a specific time point *t*. We assume the extrapolated value *I* would be obtained after *Δt*. Then its time point would be *t*+*Δt* and extrapolated value *e* could be *I*(*t*+*Δt*). So, based on equation 12, *I*(*Δt*) = *I*(*t*+*Δt*) - *I*(*t*) could be

$$I\left( \Delta t \right)=\frac{1}{\gamma}e^{-\left( 1-\theta\right)\gamma t}(1-\frac{1}{\gamma}e^{-(1-\theta)\gamma\Delta t}), \left( 15 \right)$$

Where we can figure out $\frac{1}{\gamma}I^{-\left( 1-\theta\right)\gamma t}=I\left( t \right)-\frac{1}{\gamma}$ according to equation 12. Then equation 15 can be simplified as follows:

$$I\left( \Delta t \right)=(I\left( t \right)-\frac{1}{\gamma})(1-\frac{1}{\gamma}e^{-(1-\theta)\gamma\Delta t}), \left( 16 \right)$$

So, the extrapolated value *I*(*t*+*Δt*) can be estimated from estimated parameters *θ* and *γ* and observed value *I* with a given period *Δt*. Except parameters inference and extrapolation equation building, other preprocessing, balance KNN pooling, visualization of velocity, linear regression weights and top/bottom quantiles definition are referred from RNA velocity framework.

**Dynamical model**

As RNA velocity, we use EM algorithm for dynamical model. Normally, the first step of EM algorithm is initialization of parameters *φ*. As we have obtained estimation of part parameters *φ* from steady-state model, initialization of parameters *φ* could be completed based on steady-state model. Dynamical model of one-sided loop extrusion model could be referred to Bergen’s work.

For EM algorithms of one-sided loop extrusion model, time is still a hidden variable. The parameters *φ* (*α*, *θ*, *γ*) should be infer iteratively with hidden variables *t* in a specific cell c. We can assume the difference between observed value and expected value is normal distribution in which mean value is 0 and variance is σ^2^. Then the likelihood for a TAD can be

$$L\left( \varphi\right)=\frac{1}{2\pi\sigma^{2}}exp\left( \frac{1}{n}\sum_{c}^{n} -\frac{{{(I}_{c}-I(t_{c},\varphi))}^{2}+{{(S}_{c}-S(t_{c},\varphi))}^{2}}{2\sigma^{2}} \right), \left( 17 \right)$$

where $I\left( t_{c},\varphi\right)$ and $S(t_{c},\varphi)$ are the solution of equation 11 and 12 at time *t_c_* and parameters $\varphi$ for the cell *c*. Then the log-likelihood $l\left( \varphi\right)$ have this proportionality:

$$l\left( \varphi\right)\propto-\sum_{c}^{n} {{(I}_{c}-I(t_{c},\varphi))}^{2}+{{(S}_{c}-S(t_{c},\varphi))}^{2}. \left( 18 \right)$$

So, the hidden variable *t* for different cell *c* can be solved by maximum likelihood estimation (MLE) of $l\left( \varphi\right)$:

$$t_{c}=\underset{\varphi}{\mathrm{argmax}} l\left( \varphi\right)=\underset{\varphi}{argmin(} {{(I}_{c}-I(t_{c},\varphi))}^{2}+{{(S}_{c}-S(t_{c},\varphi))}^{2}). \left( 19 \right)$$

As the derivative method will encounter the transcendental equation, in this study, *t_c_* is solved by quasi-Newton method which specifically refers to Broyden–Fletcher–Goldfarb–Shanno (BFGS) algorithm(Byrd, et al., 1995) with low memory in R package(Computing, 2013).

Parameters *φ* are still solved by BFGS. The final step of EM algorithm is the iterations of second step and third till convergence. The convergence condition in this study is set to 100 (default but user-defined) iterations or (*φ*’_iteration+1_-*φ*’_iteration_)/ *φ*’_iteration_ < 1%. In summary, the EM algorithm in this study can be stated as follow:

Step 1. Initialization of parameters *φ* (*α*, *θ, γ*) from stead-state model.

Step 2. Inference of hidden variable *h*(*t*) for each TAD from current parameters *φ*.

Step 3. Inference of parameters *φ* (*α*, *θ, γ*) for each TAD from *h*(*t*) in step 2.

Step 4. Iterations of step 2 and step3 till convergence.

The extrapolation of cell state is easier as all parameters are estimated. For a specific period *Δt*, the projected *I*(*t*+*Δt*) can be solved with equation 11 and 12 for a single TAD. Then overall velocity of a cell is comprised of all TADs in a cell. The cell fate flow can be observed from current cell point to projected cell point in embedded UMAP plot.

In summary, with the ODE equation and the steady state resolution method in hand, the derivation of the entire equation and parameters can be solved by steady state model and EM algorithm. As this velocity model exclusively utilizes Hi-C TAD features and incorporates the loop extrusion concept, we have aptly named this model Loop velocity.
