# Supplementary material for: Exploring DNA movement through the application of droplet-based high-efficiency chromatin conformation capture (DropHiChew) and loop velocity
Source: Nucleic Acids Res. 2026 Oct 29;54(20):gkag995. doi: 10.1093/nar/gkag995 (PMC13717199; doi:10.1093/nar/gkag995)
Supplement: Supplementary file 1 [file gkag995_supplemental_files.zip › Table_S1_Cost_Comparison.docx]

# Supplementary Table S1: Cost comparison of single-cell Hi-C methods

| **Method** | **Library prep cost per sample** | **Sequencing cost per sample** | **Total cost per sample** | **Cells per sample** | **Cost per cell** |
| --- | --- | --- | --- | --- | --- |
| DropHiChew | $1,794 | $348 | $2,142 | ~7,000 | ~$0.31 |
| Dip-C | $2,500 | $500 | $3,000 | ~2,400 | ~$1.25 |
| sn-m3C-seq | $3,500 | $600 | $4,100 | ~2,000 | ~$2.00 |
| sci-Hi-C | $1,200 | $400 | $1,600 | ~8,000 | ~$0.20 |

*Note: Costs are estimated based on 2024 pricing and may vary by region and facility. DropHiChew costs include 10x Genomics Chromium kit ($1,594) and library preparation reagents ($200). Sequencing costs assume 400M reads per sample at $0.87/M reads.*
