# Supplementary material for: Exploring DNA movement through the application of droplet-based high-efficiency chromatin conformation capture (DropHiChew) and loop velocity
Source: Nucleic Acids Res. 2026 Oct 29;54(20):gkag995. doi: 10.1093/nar/gkag995 (PMC13717199; doi:10.1093/nar/gkag995)
Supplement: Supplementary file 1 [file gkag995_supplemental_files.zip › Table_S2_Method_Comparison.docx]

# Supplementary Table S2: Comparison of single-cell chromatin conformation capture methods

| **Method** | **Platform** | **Valid pair ratio (%)** | **Unique valid pairs per cell** | **Cell throughput** | **Resolution** | **Cost per cell** | **Key advantages** | **Limitations** |
| --- | --- | --- | --- | --- | --- | --- | --- | --- |
| DropHiChew | 10x Genomics | ~30 | ~50K (50K reads) | High (~7,000) | 25kb-1Mb | ~$0.31 | High throughput; cost-effective; user-friendly | Limited to DpnII; GATC barcode bias |
| Dip-C | Custom | ~18 | ~80K (1.6M reads) | Low (~2,400) | 10kb-1Mb | ~$1.25 | High resolution; no enrichment bias | Low throughput; high sequencing cost |
| snHiChew | Custom | ~45 | ~100K (200K reads) | Medium (~3,000) | 10kb-1Mb | ~$2.00 | Highest valid pair ratio | Requires custom barcoding; complex workflow |
| sn-m3C-seq | Custom | ~35 | ~60K (150K reads) | Medium (~2,000) | 10kb-1Mb | ~$2.00 | Multi-omics capability | Complex protocol; low throughput |
| sci-Hi-C | Custom | ~15 | ~40K (300K reads) | Very High (~8,000) | 25kb-1Mb | ~$0.20 | Highest throughput | Lower valid pair ratio; complex barcoding |
| Droplet Hi-C | 10x Genomics | ~15 | ~30K (200K reads) | High (~5,000) | 25kb-1Mb | ~$0.50 | Commercial platform; high throughput | Lower valid pair ratio; no enrichment |

*Note: Values are approximate and based on published data and our own experiments. Valid pair ratio = non-duplicated valid pairs / total sequenced read pairs. Cell throughput values represent typical yields per experiment.*
